# 1.63-billion-year-old multicellular eukaryotes from the Chuanlinggou Formation in North China

**DOI:** 10.1126/sciadv.adk3208

**Published:** 2024-01-24

**Authors:** Lanyun Miao, Zongjun Yin, Andrew H. Knoll, Yuangao Qu, Maoyan Zhu

**Affiliations:** ^1^ State Key Laboratory of Palaeobiology and Stratigraphy, Nanjing Institute of Geology and Palaeontology, Chinese Academy of Sciences, Nanjing 210008, China.; ^2^ Department of Organismic and Evolutionary Biology, Harvard University, Cambridge, MA 02138, USA.; ^3^ Institute of Deep-sea Science and Engineering, Chinese Academy of Sciences, Sanya 572000, China.; ^4^ College of Earth and Planetary Sciences, University of Chinese Academy of Sciences, Beijing 100049, China.

## Abstract

Multicellularity is key to the functional and ecological success of the Eukarya, underpinning much of their modern diversity in both terrestrial and marine ecosystems. Despite the widespread occurrence of simple multicellular organisms among eukaryotes, when this innovation arose remains an open question. Here, we report cellularly preserved multicellular microfossils (*Qingshania magnifica*) from the ~1635-million-year-old Chuanlinggou Formation, North China. The fossils consist of large uniseriate, unbranched filaments with cell diameters up to 190 micrometers; spheroidal structures, possibly spores, occur within some cells. In combination with spectroscopic characteristics, the large size and morphological complexity of these fossils support their interpretation as eukaryotes, likely photosynthetic, based on comparisons with extant organisms. The occurrence of multicellular eukaryotes in Paleoproterozoic rocks not much younger than those containing the oldest unambiguous evidence of eukaryotes as a whole supports the hypothesis that simple multicellularity arose early in eukaryotic history, as much as a billion years before complex multicellular organisms diversified in the oceans.

## Teaser

Fossil evidence suggests that eukaryotes evolved multicellularity not long after their first appearance in Paleoproterozoic era.

## INTRODUCTION

The cell is the fundamental unit of life on Earth (*1*). The first organisms were most likely unicellular, and numerous clades still complete their life cycles as single cells. That said, multicellularity has arisen many times within bacteria and the Eukarya (*2*, *3*). Most of these clades comprise simple multicellular organisms, with cell-cell adhesion but limited communication or differentiation among constituent cells; complex multicellularity, with greater directed intercellular communication and more pronounced cell and tissue differentiation, has arisen only six to seven times, all within the Eukarya (*2*, *3*).

The evolution of multicellularity is a question of history and process, and paleontological records can potentially tell us when and under what conditions multicellular eukaryotes first evolved. Fossils found on several continents show that in the oceans, simple multicellular eukaryotes, such as uniseriate filaments and coenobia, arose long before the advent of complex multicellular animals and algae (*2*, *3*); prokaryotic multicellularity extends even further back into the Archean (*4*). Relatively abundant late Mesoproterozoic to early Neoproterozoic populations include forms interpreted as red [*Bangiomorpha pubescens*, ~1050 million years (Ma), arctic Canada (*5*, *6*)] or green [*Proterocladus antiquus*, ~950 Ma, North China (*7*)] algae, as well as putative early fungi [*Ourasphaira giraldae*, ~890 Ma, Arctic Canada (*8*)] and eukaryotic problematica, including *Eosolena loculosa* [~1030 Ma, Siberia (*9*)], *Arctacellularia tetragonala* [~1000 Ma, Congo (*10*)], and *Archaeochaeta guncho* [~950 Ma, northwestern Canada (*11*)]. Less common records of both cellularly preserved microfossils such as *Eosolena minuta* from northern Siberia (*12*) and decimeter-scale carbonaceous compressions from North China (*13*) extend the record of eukaryotic multicellularity back to the early Mesoproterozoic era. If their stratigraphic placement is correctly interpreted, then phosphatized microfossils from India (*14*) would add substantially to this record. The coiled ribbon–like macrofossil *Grypania* from India not only shows clear evidence of multicellularity and large size consistent with a eukaryotic affinity (*3*), but it also has been interpreted as a possible giant cyanobacterium (*15*). Other coeval (*16*) and older records (*17*) of *Grypania* are more controversial because of the absence of preserved cellular structure, much like pyritic macrostructures reported from ~2.1-Ga shales in Gabon (*18*) and carbonaceous compressions from the ~1630-Ma Tuanshanzi Formation in North China (*19*), whose biological origins are uncertain.

Large uniseriate filaments described as *Qingshania magnifica*, with cell diameters up to 250 μm, were described as early as 1989 by Yan (*20*) from thin sections of shales within the late Paleoproterozoic Chuanlinggou Formation, North China, and interpreted as primitive green algae. Owing to the poor image quality of the material described and its publication in a relatively difficult-to-access journal, this report has received little attention since its publication. Here, we revisit the question of Paleoproterozoic multicellularity and report abundant, organically preserved multicellular filaments extracted from shales of the Chuanlinggou Formation in the Yanshan Range, North China. Their overall morphology and size range, as well as stratigraphic position, suggest that these filaments can be assigned to *Q. magnifica*. Our materials reveal conspicuous morphological details and additional characters (e.g., spheroidal intracellular structures), which, in combination with spectroscopic evidence, provide strong support for the interpretation of these fossils as eukaryotic, thus indicating that eukaryotes evolved simple multicellularity and likely, photosynthesis, early in the history of the domain.

## RESULTS

### Materials and age constraint

The microfossils were isolated by acid maceration from dark gray shales ~100 m below top of the Chuanlinggou Formation in the Wengjiazhuang section (40°35′15″N; 118°32′15″E), Kuancheng County, Hebei Province (fig. S1). The Chuanlinggou Formation forms the middle portion of the ~2700-m Changcheng group in the Yanshan Range, North China Craton (NCC). Underlain by sandstones of the Changzhougou Formation and overlain by Tuanshanzi and Dahongyu carbonates, Chuanlinggou strata in the Kuancheng area comprise an ~400-m-thick succession of mostly shallow marine shales and siltstones, with intercalations of thinly bedded dolostone in their upper part (*21*). Specifically, shales harboring microfossils in upper Chuanlinggou Formation have been interpreted as shallow subtidal deposits (*22*). The age of microfossils is well constrained by an ash bed ~40 m above the fossil horizon in the Kuancheng area, which has yielded a U-Pb zircon age of 1634.8 ± 6.9 Ma (*23*).

### Microfossil morphology

**Fig. 1.**
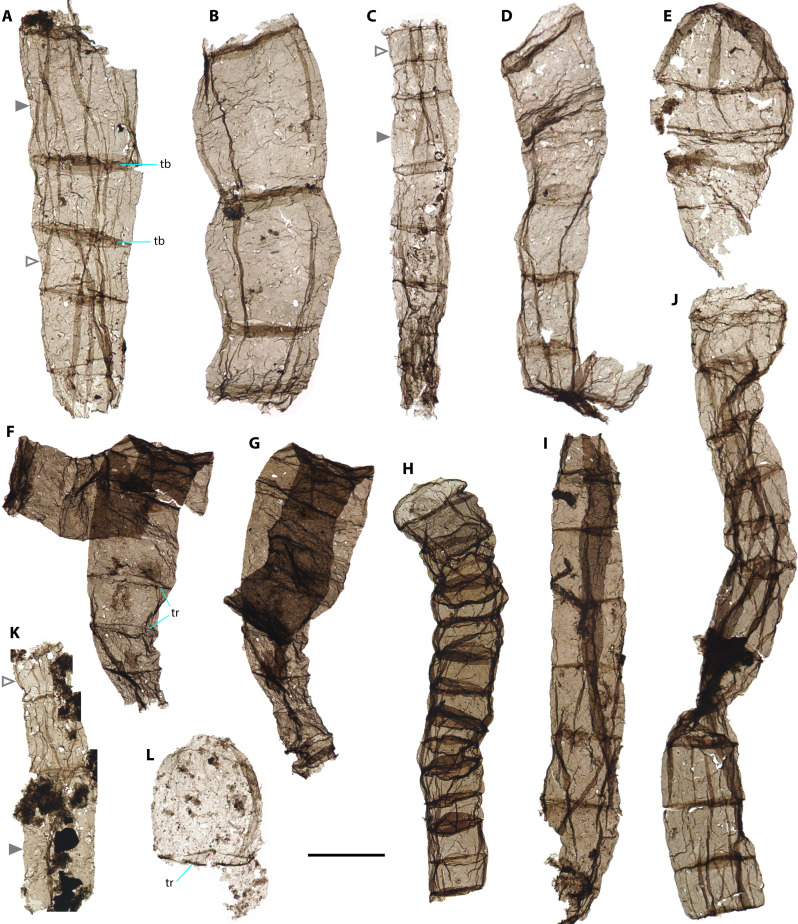
Transmitted-light (TL) photomicrographs of *Q. magnifica* from the Chuanlinggou Formation. (**A** to **D** and **K**) Filaments with cells of varying length and width. (**E**) Four-celled filament with hemispherical terminal cell. (**F** and **G**) Filament with notably decreasing cell width toward one end. Note that (F) and (G) represent the same specimen; (F) lost the narrowest part of the filament as shown in (G). (**H** to **J**) Filaments displaying more uniformity of cell dimensions. (**L**) Two-celled filament with ovoid terminal cell. All specimens were handpicked from organic residues of acid maceration and photographed in wet mounts, except for (K), which was photographed from a permanent strew mount. Solid and empty gray triangles in (A), (C), and (K) indicate the longest and the shortest cells, respectively, within single filaments. tb, transverse band (interpreted as cross wall); tr, transverse ring (interpreted as partially preserved cross wall). Scale bar, 50 μm [(A) to (E), (I), (J), and (L)] and 100 μm [(F) to (H) and (K)].

**Fig. 2.**
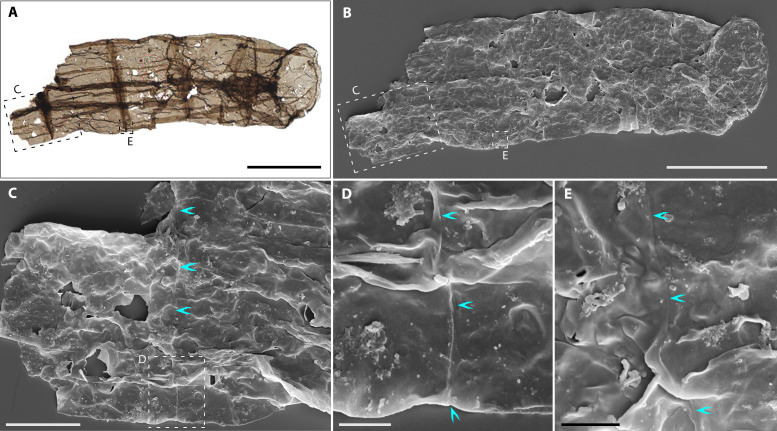
Micrographs of *Q. magnifica* from the Chuanlinggou Formation. (**A**) TL photomicrograph of a five-celled filament with constant width and dark narrow transverse bands. (**B**) SEM image of (A) showing surface features and the preservation as a complete compression. Note the obliquely compressed cross wall of the right terminal cell showing smooth surface and no other particular features. (**C** to **E**) Magnifications of (B), showing smooth wall surface and the well-defined contact between adjoining cells manifested by a very shallow groove (marked by cyan arrowheads) along transverse bands. (C) and (E) represent dashed boxes in (A) and (B); (D) corresponds to the dashed box in (C). Scale bars, 50 μm [(A) and (B)], 10 μm (C), and 2 μm [(D) and (E)].

**Fig. 3.**
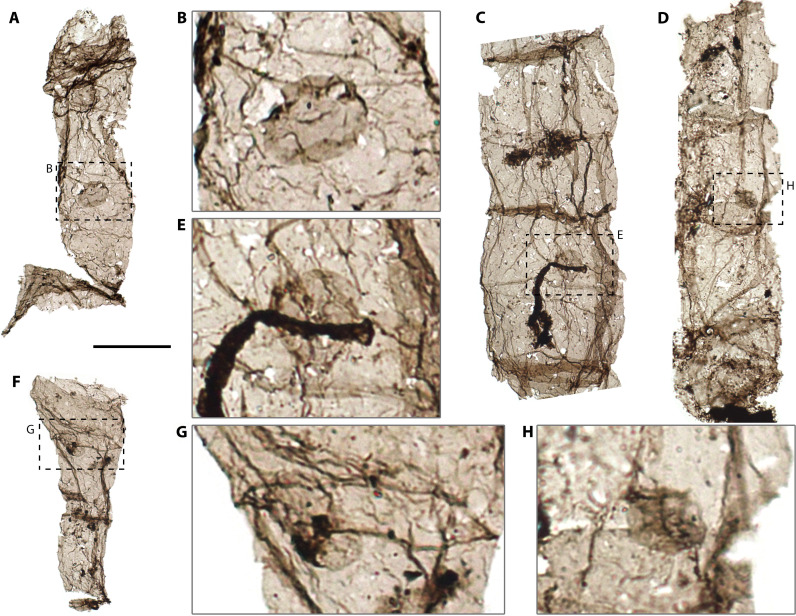
TL photomicrographs of *Q. magnifica* with a small round or ovoid inclusion from the Chuanlinggou Formation. (**A**, **C**, and **D**) Filaments with constant width. (**B** and **E**) Magnifications of dashed boxes in (A) and (C), respectively, showing details of round inclusions. (**F**) Filament of notably varying width. Note that the middle cell of the filament is cyathiform in shape. (**G** and **H**) Magnifications of dashed box in (F) and (D), respectively. All specimens were handpicked from organic residues of acid maceration and photographed in wet mounts. Scale bar, 50 μm [(A), (C), (D), and (F)].

**Fig. 4.**
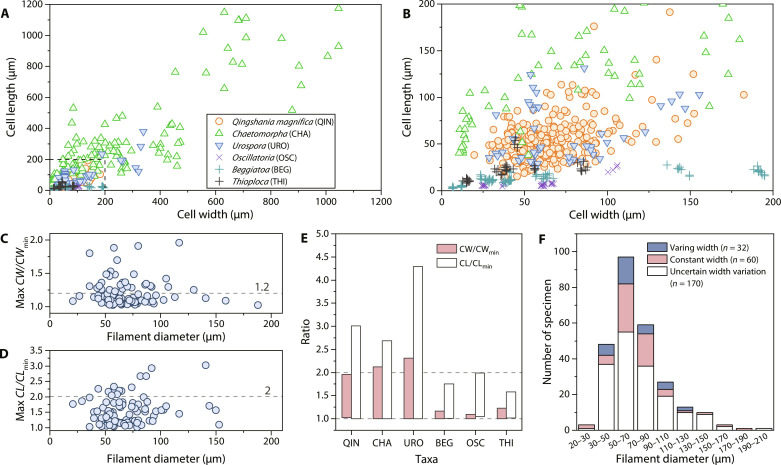
Morphometric analyses of *Q. magnifica* from the Chuanlinggou Formation. (**A** and **B**) Scatter plot of cell length and cell width of *Q. magnifica* along with those of selected extant eukaryotic algae and filamentous bacteria. (B) Magnification of the dashed box in (A). (**C**) Scatter plot of maximum ratio of cell width to minimum cell width (CW/CW_min_) within single filaments of *Q. magnifica*. Filament with constant width has ratio close to 1. Filament with ratio > 1.2 is interpreted with confidence as having varying width. Filaments with ratio in between represent transitional forms, here grouped with filaments having constant width, with consideration of taphonomic influence and measurement error. (**D**) Scatter plot of maximum ratio of cell length to minimum cell length (CL/CL_min_) within single filaments of *Q. magnifica*. Note that a small number of specimens have ratio > 2, suggesting large variation in cell length within single filament. (**E**) Grouped floating bar chart showing ratios of CW/CW_min_ and CL/CL_min_ within single filaments of *Q. magnifica* and the selected extant eukaryotic algae and filamentous bacteria. Abbreviations of taxa are provided in the legend of (A). (**F**) Stacked column chart showing size-frequency distribution of *Q. magnifica*. Measurements and frequency data of *Q. magnifica* are provided in data S1. Size data of green algae *Chaetomorpha* and *Urospora*, cyanobacteria *Oscillatoria*, and sulfur bacteria *Beggiatoa* and *Thioploca* were measured from scaled illustrations in the literature and are provided in table S1 along with cited references.

Recovered filaments occur as pale to grayish brown, flattened translucent fragments (Figs. 1 to 3), including specimens in petrographic thin sections (fig. S2A). This state of preservation demonstrates their syngenicity with enclosing shales. The microfossils are described here as *Q. magnifica* and can be differentiated from other large filamentous taxa in both morphology and size (for details see the “Systematic paleontology” section). From a sample population of 278 microfossils, we derived the following description: (i) unbranched, uniseriate filaments have cross-sectional diameter of 20 to 194 μm (mean = 73 μm; SD = 29 μm; *n* = 262) and length up to 860 μm (Fig. 4B); (ii) filaments are mostly straight or occasionally curved, comprising a few to more than 20 smooth-walled cells; (iii) cells are chiefly cylindrical, without notable constrictions at cell-cell boundaries. Cell length is of 15 to 190 μm, with a width/length ratio of 0.48:3. Terminal cells shown by two short filaments are hemispherical (Fig. 1E) or ovoid (Fig. 1L); (iv) wall thickness is of ~0.15 μm, measured from walls around taphonomic holes in scanning electron microscope (SEM) images (Fig. 2); (v) cross-walls (septa) are prominent and preserved as dark, narrow transverse bands seen via transmitted light microscopy (TLM) (e.g., Fig. 1A) or indicated by transverse rings outlined by dark lines of ~1-μm-thick when poorly preserved (e.g., Fig. 1, F and L). The cross-wall surface is smooth and shows no distinctive features, indicated by an obliquely compressed specimen (Fig. 2, A and B). No external sheath or holdfast structure was observed (Fig. 2 and fig. S3).

Despite their simple and consistent organization, the filaments display a moderate degree of morphological variation in cell shape and size, both within and among filaments. Individual cells can be cylindrical, barrel-shaped (e.g., Fig. 1, A and B), or cyathiform (Fig. 3F). Filaments may be constant in width (Figs. 1J and 2A), taper toward one end throughout the preserved specimen (Figs. 1, A to D and K, and 3F), or taper only at or near its terminus (Fig. 1, F and G). Despite this morphological variability, there are no distinct subpopulations observed in morphometric data (Fig. 4), suggesting that they were probably derived from a single biological species. Their large size range, in which the widest can be 10 times larger than the narrowest, likely reflects different growth or developmental stages within the population.

In addition, a small round to ovoid structure with a diameter of 15 to 21 μm is found in association with a few filaments (4 of 278 specimens; Fig. 3). The structure is thin, only faintly visible against its host filament under TLM, and occurs one per cell at the center or near a cross wall; in all cases, the structures occur entirely within the boundaries of filament cells and do not overlap with cell walls. They show the same focal depth with respect to associated filaments under TLM and exhibit a notable uniformity in size, number, locus, shape, texture, and transparency across all observed specimens (Fig. 3). These features—combined with the uniform thickness (as judged by uniform transparency), smooth surface, even edges, and well-defined shape—indicate that the structures were originally thin-walled biological structures within individual cells. It is highly improbable that they represent superimposed spheroids, as randomly overlapping structures are unlikely to display this consistency. Spheroidal fossils are relatively rare in this fossil assemblage, the only three specimens observed being larger (45 to 75 μm) than the intracellular spheroids. Moreover, solitary spheroids comparable in size to the intracellular structures have not been observed from other upper Chuanlinggou samples.

Intracellular inclusions (ICIs) in Proterozoic and Phanerozoic cells have been variously interpreted as reproductive structures [e.g., endocysts (*24*)], collapsed cytoplasm (*25*), or organelles [e.g., nuclei (*26*), plastids (*25*), and pyrenoids (*14*)]. Given that the structures observed here are compressed cell walls such as the inferred endocysts of microfossils from Ediacaran and Phanerozoic shales (*24*), the latter interpretations can be excluded for our specimens. Similar structures in extant organisms include asexual spores found within multiple algal lineages [e.g., aplanospores of *Klebsormidium* (Charophyceae) (*27*), *Tribonema* (Xanthophyceae) (*28*), and *Uronema* (Chlorophyceae) (*29*)], endospores of some bacteria [e.g., *Bacillus anthracis* (*30*)], and endoconidia (asexual spores) of some fungi [e.g., *Thielaviopsis basicola* (*31*)], functioning in reproduction and also as a survival strategy similar to some bacterial endospores. An endospore is produced in response to nutrient depletion or other adverse environmental conditions and characteristically has a thick protective envelope consisting of several proteinaceous layers (*30*). The ICIs here have very thin walls as indicated by their faint appearances under TLM (Fig. 3), so a dormant endospore does not represent a likely functional analog. Therefore, the ICIs mostly likely represent reproductive structures, such as asexual spores. That their size is only slightly smaller than that of the narrowest filaments is consistent with this interpretation. Similar structures can be seen in some extant filamentous algae [e.g., *Urospora wormskioldii* (*32*)]. We noted the rarity of ICIs in the fossil assemblage, which does not conflict with the interpretation as spores. There are many reasons why spores might not be abundant in associated sediments. For example, reproduction might be seasonal or induced by specific environmental conditions; moreover, reproduction only occurs in certain stages in the life cycle, and spores would be released into the environment upon reaching maturity.

### Chemical characterization of microfossils

In addition to morphological analysis, we performed microscale Raman and Fourier transform infrared (FTIR) spectroscopic investigations to characterize the chemical composition of *Q*. *magnifica* to better constrain its biological affinity. For comparison, analyses were conducted on three co-occurring cyanobacterial taxa, i.e., *Oscillatoriopsis princeps* (fig. S2C), *Siphonophycus punctatum* (fig. S2D), and *Pseudodendron* sp. (fig. S2E), from a single shale sample same as *Q*. *magnifica*.

**Fig. 5.**
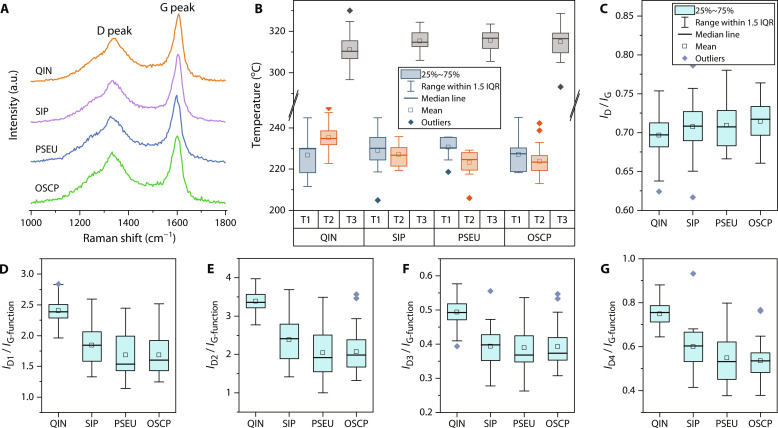
Raman analyses of *Q. magnifica* and co-occurring cyanobacterial microfossils from the Chuanlinggou Formation. (**A**) Representative Raman spectra of the first order region with baseline corrected and intensity normalized (from 0 to 1). (**B**) Box-and-whisker plot of peak metamorphic temperatures estimated by Raman parameters. T1 to T3 represent temperature calculated on the basis of Raman reflectance (*33*), FWHM-D1 and FWHM-D2 (*34*), respectively. Note that T1 (205° to 245°C) and T2 (206° to 251°C) highly overlap with each other across all analyzed specimens, representing reasonable estimates; whereas T3 (293° to 330°C) is far beyond the suggested temperature range (50° to 200°C) for this parameter (*34*), thus representing a problematic estimate. (**C**) Box-and-whisker plot of the intensity ratio of D to G peak (*I*_D_/*I*_G_). (**D**) Box-and-whisker plot of intensity ratio of D1 to G-function band (*I*_D1_/*I*_G-function_). (**E**) Box-and-whisker plot of intensity ratio of D2 to G-function band (*I*_D2_/*I*_G-function_). (**F**) Box-and-whisker plot of intensity ratio of D3 to G-function band (*I*_D3_/*I*_G-function_). (**G**) Box-and-whisker plot of intensity ratio of D4 to G-function band (*I*_D4_/*I*_G-function_). Analyzed specimens are illustrated in fig. S2. QIN, *Q. magnifica*; SIP, *S. punctatum*; PSEU, *Pseudodendron* sp.; OSCP, *O. princeps*. Source data are provided in data S2. a.u., arbitrary units.

**Fig. 6.**
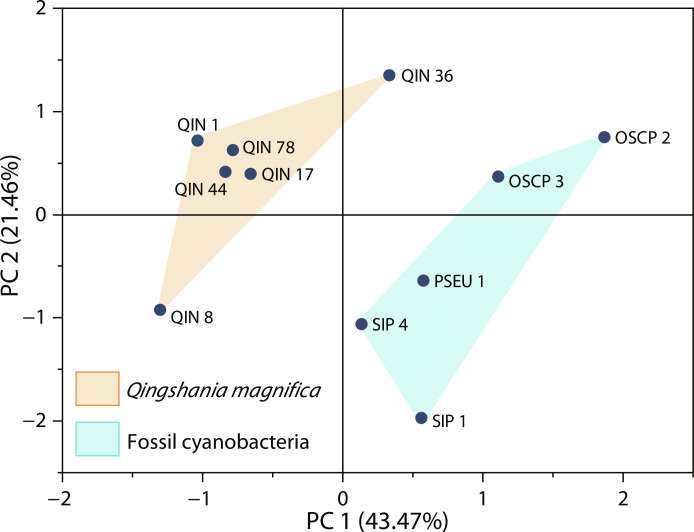
Score plot for PCA of *Q. magnifica* and co-occurring cyanobacterial microfossils from the Chuanlinggou Formation. Abbreviations of taxa correspond to those in Fig. 5. Source data are provided in data S2.

The Raman spectra of *Q*. *magnifica* show two peaks at ~1350 cm^−1^ (D peak) and ~1600 cm^−1^ (G peak), indicating typical disordered carbonaceous material, which are similar to those of the cyanobacteria (Fig. 5A). Using Raman parameters [Raman reflectance (*33*) and the full width at half maximum (FWHM) of D1 subpeak (*34*)], peak metamorphic temperature was estimated to be 205° to 250°C, with an average of ~230°C (Fig. 5B), suggesting that all fossils experienced low-grade metamorphism. This is in agreement with their highly convergent intensity ratio of D to G peaks (*I*_D_/*I*_G_) (Fig. 5C), which has been used to assess the structural order of organic matter (*35*). Despite the substantial thermal alteration, organic remains of *Q*. *magnifica* show unambiguous differences from those of cyanobacteria, as indicated by the principal components analysis (PCA) of Raman spectra. In the PCA, *Q*. *magnifica* show distinct deviation from cyanobacterial fossils (Fig. 6); the latter were clustered together, suggesting their highly similar chemical composition. The principal component (PC) 1 accounts for the separation (43.47% of total variance). The PC loading (fig. S4A) shows that spectral regions of 1150 to 1320 cm^−1^ and 1550 to 1630 cm^−1^ provide the greatest contribution to the PC.

To better distinguish the structural characteristics, Raman spectra were deconvoluted into five subpeaks, D1 (~1350 cm^−1^), D2 (~1610 cm^−1^), D3 (~1510 cm^−1^), D4 (~1245 cm^−1^), and G functions (~1580 cm^−1^) (fig. S4B), following a widely used fitting protocol (*34*). Among them, G function is attributed to in-plane stretch (*E*_2g_ mode) of aromatic layers in graphite (*36*). D1 and D3 functions are related to structural defects and heteroatoms within and outside the plane of aromatic layers, respectively (*36*). D2 is also introduced by disorders (intravalley defects) in aromatic structures (*37*) and merges with G function to form a single band at 1600 cm^−1^ (the general G peak) in low-ordered carbonaceous matter (*38*). D4 is associated with vibrations of aliphatic hydrocarbon chains (*39*). Because D functions represent the disordered carbonaceous component, the intensity ratio of D versus G functions may also be used to evaluate the structural order of carbonaceous material, ranging from disordered amorphous carbon to well-crystallized graphite. As shown in Fig. 5 (D to G), *Q*. *magnifica* exhibits relatively higher ratios (*I*_D1_/*I*_G-function_, *I*_D2_/*I*_G-function_, *I*_D3_/*I*_G-function_, and *I*_D4_/*I*_G-function_) than those of cyanobacteria taxa, indicating a more disordered molecular structures within fossilized organic remains. In addition, consistent with PCA, these ratios (Fig. 5, D to G) of cyanobacterial taxa are more convergent with each other. As the microfossils experienced the same post-depositional processes, the spectral differences observed between *Q*. *magnifica* and cyanobacterial taxa most likely reflect differences in precursor molecular compositions.

**Fig. 7.**
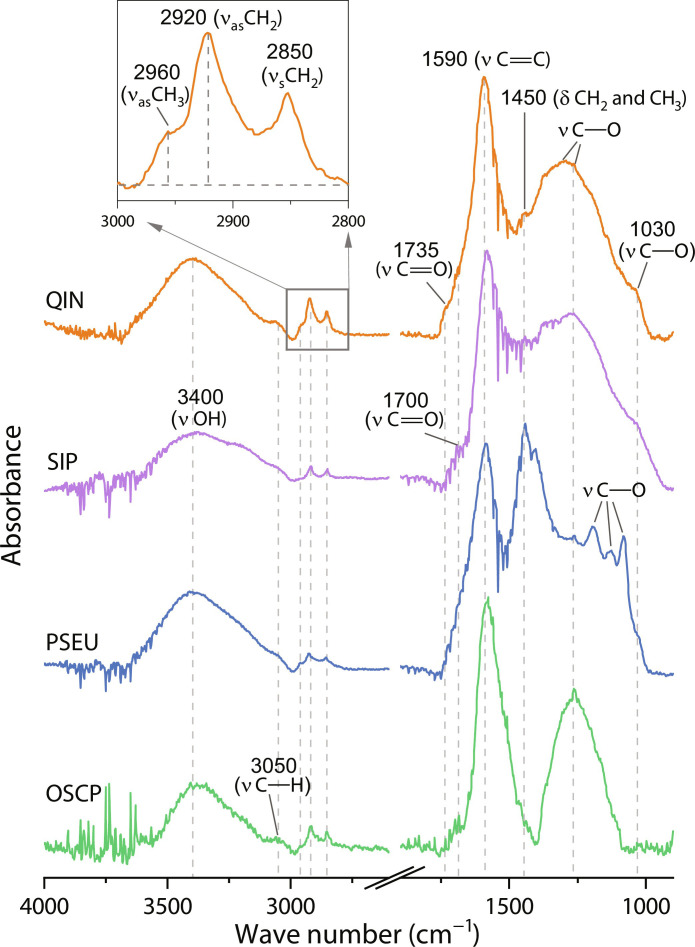
Representative FTIR spectra of *Q. magnifica* and co-occurring cyanobacterial microfossils from the Chuanlinggou Formation. Spectra were baseline corrected and normalized in intensity from 0 to 1. The insert shows the aliphatic bands in the 3000 to 2800 cm^−1^ region. Band assignments and vibration modes (δ, deformation; ν, stretching; s, symmetric; as, asymmetric) are provided in table S2. Analyzed specimens are illustrated in fig. S2. Abbreviations of taxa correspond to those in Fig. 5. Source data are provided in data S2.

FTIR data provide further information about functional groups in the organic constituents of the microfossils. Spectra of *Q*. *magnifica* show several absorption bands (Fig. 7 and table S2), including the most prominent band at ~1590 cm^−1^ (C═C ring breathing); a broad band at ~3400 cm^−1^ (hydroxyl O─H stretch); a moderate band at ~1450 cm^−1^ (methyl CH_3_ and methylene CH_2_ bend); some weak bands as shoulders at ~3050 cm^−1^ (aromatic = C─H stretch) and ~1735 and ~1700 cm^−1^ (carbonyl C═O stretch) and a few bands in the 1300- to 1000-cm^−1^ region (aromatic or aliphatic C─O stretch); and three weak aliphatic bands at ~2960 cm^−1^ (CH_3_ asymmetric stretch), ~2920 cm^−1^ (CH_2_ asymmetric stretch), and ~2850 cm^−1^ (CH_2_ symmetric stretch) (*40**–**42*). The presence of intense aromatic absorption and weak aliphatic bands in 3000- to 2800-cm^−1^ region suggest that the organic matter was dominated by aromatics with low aliphatic compounds. In addition, the chemometric ratio R_3/2_ (intensity ratio of CH_3_ at ~2960 cm^−1^ to CH_2_ at ~2920 cm^−1^) is often used as an index for evaluating the degree of branching and chain length of aliphatic structures (*42*). The R_3/2_ ratio of *Q*. *magnifica* has a low value of 0.34 ± 0.01, which indicates the presence of long and less branched aliphatic carbon chains in the organic matter. FTIR spectra for the fossil cyanobacteria contain absorption bands similar to those of *Q*. *magnifica* but with differences in absorption intensity of some bands (e.g., aliphatic bands) and the absence of carbonyl C═O absorption in ester at ~1735 cm^−1^ (Fig. 7). In short, while spectroscopic analyses do not by themselves permit unambiguous phylogenetic placement of *Q. magnifica*, they do indicate that it is distinct from known cyanobacteria in the same beds.

## DISCUSSION

*Q. magnifica* Yan, 1989 (*20*) was first reported from the upper Chuanlinggou Formation in the Jizhou area (previously known as Jixian County; fig. S1C), North China. The type materials, identified from thin sections of yellowish-green shales, exhibited large sizes up to 250 μm in width and 6000 μm in length. The fossils were interpreted as primitive green algae and placed in the family Ulotrichaceae (*20*). Our specimens isolated from equivalent shales in the Kuancheng area (fig. S1C) reveal detailed morphological features and additional traits such as ICIs, permitting enhanced morphological reconstruction. *Q. magnifica* can be recognized as simple multicellular organisms characterized by large size and moderate morphological variation, as well as a life cycle that included reproduction via spores that were differentiated intracellularly and an apparent pattern of growth from thin to thick filaments.

In the extant biota, organisms with simple multicellularity are widely distributed among both prokaryotic (*43*, *44*) and eukaryotic lineages (table S3) (*45*, *46*). More than 140 genera (within 11 bacterial and 1 archaeal phyla) of present-day filamentous prokaryotes are known to exhibit obligate or facultative multicellularity (fig. S5 and table S4). Among them, cyanobacteria are of particular interest in that they are known to preserve well in the fossil record and can have relatively large size, with filaments ranging from simple forms with undifferentiated cells of uniform diameter [e.g., *Oscillatoria* (*47*)], filaments that taper apically [e.g., *Rivularia* (*48*)], and complex forms with true branches and cell differentiation [e.g., *Fischerella* (*49*)]. None of these, however, compare closely with *Q. magnifica* in terms of size, the absence of an external sheath or inferred reproductive mode. Although some *Oscillatoria* (*47*) and *Lyngbya* (*50*) species may form filaments up to 100 μm in diameter, they are markedly different from *Q. magnifica* in form made up of undifferentiated disc-shaped cells. Moreover, within single species, there is no notable size difference among trichomes due to their reproduction by hormogonia or simple breakage of filaments. The same is true for other large bacteria, such as sulfur bacteria *Beggiatoa* (*51*) and *Thioploca* (*52*), which are morphologically similar to large cyanobacteria. Our Raman and FTIR analyses show that the organic matter composition of *Q*. *magnifica* is different from that of co-occurring cyanobacterial taxa (Figs. 5 to 7), further excluding the possibility of a cyanobacterial origin. Other prokaryotic lineages (fig. S5 and table S4) are characteristically very small, with diameters up to three orders of magnitude smaller than *Q. magnifica*. These include the endospore-forming bacteria, e.g., *Bacillus* (*30*) and segmented filamentous bacteria (*53*). However, their tiny sizes fail to support a reliable comparison to *Q. magnifica*. Some bacteria, e.g., *Thiomargarita magnifica* (*54*), can reach macroscopic size up to 2 cm in length, but these large vacuolated organisms are unicellular.

Overall, prokaryotic filaments can have large cells, relatively complex morphology, multicellularity, or inner spores, but none are known to combine all of these characters. In contrast, the suite of features observed in *Q. magnifica* can be found in a number of extant eukaryotic algae [e.g., *Urospora* (*55*) and *Chaetomorpha* (*56*)], allowing a confident assignment to the eukaryotic domain. As an example, *Q. magnifica* compares favorably with the green alga *Urospora* in overall morphology, cell shape (cylindrical and barrel-shaped), cell size distribution (Fig. 4, A and B), and the pattern of cell size variation within single filaments (Fig. 4E). In addition, some species of *Urospora* show large cell size range during their life cycle in which the largest can be 10 to 20 times wider than those of the youngest filaments (*32*), similar to that observed in *Q. magnifica*. However, *Urospora* has complex life histories involving a codiolum stage and reproduce by both sexual reproduction and asexual zoospores or aplanospores. Further detailed comparison is impossible because of the incomplete preservation of *Q. magnifica*.

Extant uniseriate filamentous eukaryotes include diverse photosynthetic algae in both the Archaeplastida and Ochrophyta, septate filamentous fungi, and oomycetes (table S3), all having semirigid cell walls. The presence of cell walls that completely surround individual cells of *Q. magnifica* suggest that these organisms gained nutrition either by photosynthesis or osmotrophy (*57*). The septate hyphae of osmotrophic organisms such as *Neurospora crassa* (*58*) may show some broad morphological similarity to *Q. magnifica*, but they differ in their relatively complex morphology, including extensive branches and mycelial networks. Nearly all known septate hyphae are thin, with diameters commonly of several to (rarely) more than 30 μm (*45*, *59*); these narrow dimensions are crucial for the large surface area–to–volume ratio needed for effective nutrient absorption. Although some fungi (*31*) may form endoconidia within hyphae, no living fungi compare well with *Q. magnifica* in terms of morphology, size, and reproduction. In addition, most molecular clock estimates point to an emergence of oomycetes near the Proterozoic-Phanerozoic boundary (*60*) or later (*61*) and a relatively late origin of fungi at about one billion years ago (*62*) [but see (*63*)]. Moreover, fossils and phylogenies both indicate that fungi with complex mycelia radiated in concert with land plants during the Paleozoic era (*64*).

**Fig. 8.**
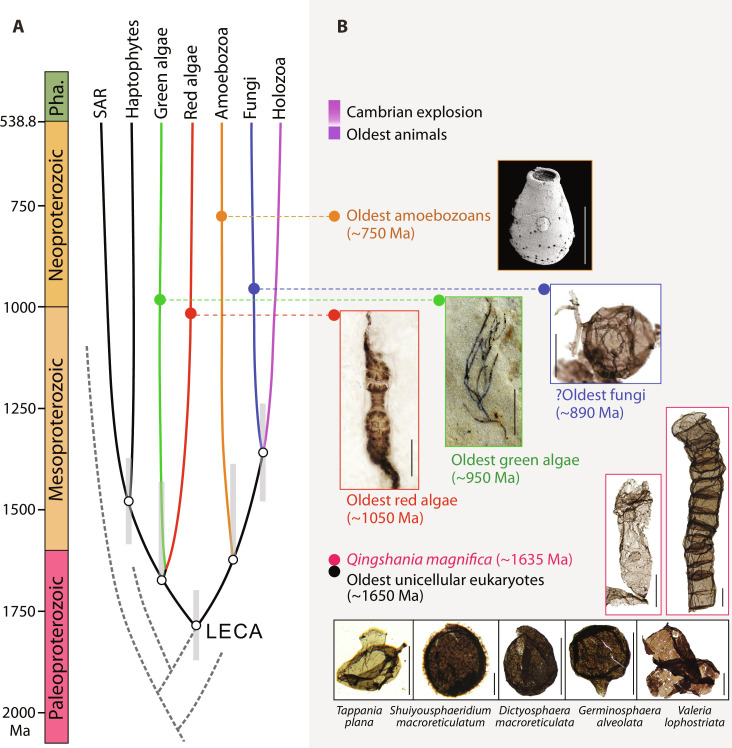
Overview of early evolution of the Eukarya along with fossil records. (**A**) Simplified eukaryotic tree with divergence time estimates of major branches by molecular clock study. LECA, last eukaryotic common ancestor. Dashed gray lines represent hypothetical stem-group eukaryotes, which are extinct. Tree topology and molecular clock estimates are from (*67*). Abbreviation: Pha., Phanerozoic. (**B**) Representative fossil records of early eukaryotes. The oldest unambiguous eukaryotic fossils are unicellular forms, e.g., *Tappania plana* and *Shuiyousphaeridium macroreticulatum* from ~1650-Ma Ruyang Group [images courtesy of L. Yin, reprinted from (*75*) with permission from Elsevier]; *Dictyosphaera macroreticulata*, *Germinosphaera alveolata*, and *Valeria lophostriata* from the Changzhougou Formation and lowermost Chuanlinggou Formation in North China [reprinted from (*76*) with permission from Elsevier]. The *Q. magnifica* represents the current oldest convincing multicellular eukaryote from ~1635-Ma upper Chuanlinggou Formation in North China. The oldest red alga is *Bangiomorpha pubescens* from ~1050-Ma Hunting Formation, Canada [image courtesy of N. Butterfield, reprinted from (*5*) Cambridge Univ. Press, reproduced with permission]. The oldest green alga is *P. antiquus* from ~950-Ma Nanfen Formation in North China [image courtesy of Q. Tang, reprinted from (*7*) with permission from Springer Nature]. The oldest putative fungus is *O. giraldae* from ~890-Ma Grassy Bay Formation in Canada [image courtesy of C. Loron, reprinted from (*8*) with permission from Springer Nature]. The oldest amoebozoans are vase-shaped microfossils, e.g., *Cycliocyrillium torquata* from ~750 to 730 Ma Kwagunt Formation, Chuar group in Arizona [image courtesy of S. Porter, reprinted from (*79*) Cambridge Univ. Press, reproduced with permission]. Scale bars, 500 μm (the image of the oldest green algal fossil equals) and 50 μm (the rest).

Therefore, to the best of our knowledge, eukaryotic algae (e.g., *Urospora*) present the best modern analogs for *Q. magnifica*. For these reasons, we infer that *Q. magnifica* was most likely photosynthetic, an interpretation congruent with molecular clock studies, which estimate that the initial acquisition of plastids occurred during the Paleoproterozoic era (*65**–**67*). An algal affinity for *Q. magnifica* is also consistent with similar but younger fossils interpreted as green algae, e.g., ~950 Ma *P. antiquus* (*7*), ~820 Ma *Proterocladus major* (*68*), and early Devonian microfossils (*25*). The Devonian filaments, in particular, show notable similarity to *Q. magnifica* in preservation and morphological details, including smooth surface, cross walls (preserved as transverse bands and transverse rings seen under TLM), cylindrical and barrel-shaped cells, and large cell size (40 to 70 μm in diameter) (*25*). They were compared with modern ulotrichalean or cladophoralean algae. Despite these similarities, the precise phylogenetic affinities of *Q. magnifica* remain uncertain, given both its great age and the absence of diagnostic features. If *Q. magnifica* were truly photosynthetic, it could represent extinct stem group Archaeplastida, stem group red or green algae, or, conceivably, an extinct eukaryotic lineage. In any event, these fossils provide support for a late Paleoproterozoic appearance of crown group eukaryotes (*68*) rather than a late Mesoproterozoic origin (*69*), as the origin of primary plastids postdated the emergence of the last eukaryotic common ancestor (Fig. 8) (*70*). Sterane biomarkers diagnostic for extant eukaryotic clades have been identified only in Neoproterozoic and younger sedimentary rocks (*71*) [but see (*72*)], but, recently, Brocks *et al.* (*73*) have found protosteroids in 1640 to 800-Ma rocks that they interpret as the products of stem group eukaryotes or stem group members of extant eukaryotic clades. Thus, the emerging biomarker record is consistent with potential phylogenetic interpretations of *Q. magnifica*.

Regardless of their phylogenetic placement, our fossils provide strong evidence that integrated multicellularity, with its underlying molecular mechanisms for cell-to-cell adhesion and communication, evolved early in eukaryote history, as did life cycles with differentiated reproductive cells (Fig. 8). The oldest unambiguous evidence for eukaryotes are unicellular fossils found in rocks not much older than those examined here (*74**–**76*). There may well be an older prehistory of eukaryotes that did not form preservable walls or inhabit environments likely to be recorded by sediments. While simple multicellular eukaryotes evolved early, complex multicellular organisms did not appear for another billion years (Fig. 8). Therefore, while simple multicellularity may be necessary for the evolution of complex multicellular organisms, it is not sufficient. Overall, by the end of the Paleoproterozoic era, the Eukarya had begun the processes of taxonomic, morphological, and metabolic diversification, which would eventually produce the world we see today (Fig. 8).

### Systematic paleontology

Genus *Qingshania* Yan, 1989, emend

Type species. *Q. magnifica* Yan, 1989 (*20*).

Emended diagnosis. Large unbranched uniseriate filaments with moderate morphological variation and diameter less than 300 μm. Filament uniform in width or tapering toward one end. Cell shape ranging from short to long cylindrical, sometimes barrel-shaped or cyathiform. Constrictions at cell interfaces commonly absent. Cells of filaments sometimes preserved with one ICI. The inclusion is translucent, round to ovoid in outline, with size up to one-half of the host cell. Sheath absent.

Discussion. The genus *Qingshania* is emended here to include features such as ICIs (Fig. 3) and filaments with constant width. It closely resembles the genus *Eosolena* [established from ~1030-Ma Neruyen Formation, Lakhanda Group in Siberia (*9*, *77*)] in general morphology and organization but differs in its smaller sizes, as type materials of the latter exhibit much larger diameters of 200 to 800 μm (*77*). Moreover, *Qingshania* has well-defined, spheroidal ICIs that occurred singly in some cells of filaments (Fig. 3), which are reminiscent of aplanospores in some extant filamentous algae [e.g., (*29*)]. Although *Eosolena* was also reported to have spheroidal structures preserved with some filaments, these structures were tiny (2 to 10 μm in diameter) and interpreted to be likely photosynthetic algal endosymbionts (*9*). However, on the basis of their dark and opaque appearance and wide occurrences in other fossil taxa in type materials (*9*), a taphonomic origin cannot be easily excluded.

*Q. magnifica* Yan, 1989, emend

(Figs. 1 to 4 and figs. S2, A and B, and S3).

Holotype. Specimen illustrated in Plate I, Fig. 1 in Yan (*20*).

Emended diagnosis. As for the genus, filament with smooth wall surface and diameter greater than 20 μm.

Discussion. *Q. magnifica* forms a population distinct from other large filamentous taxa [e.g., *E. loculosa* (*77*), *E. minuta* (*12*), *Segmentothallus asperus* (*78*), *Rafatazmia chitrakootensis* (*14*), and a Devonian filamentous alga (*25*)] as revealed by morphometric analyses (fig. S6). It also differs in having translucent ICIs (Fig. 3). More specifically, *E. loculosa* and *S. asperus* all initially described from late Mesoproterozoic Lakhanda Group (*77*, *78*) are much larger in dimension than *Q. magnifica*; *E. minuta* has constituent cells primarily short cylindrical in shape (*12*); *R. chitrakootensis* (*14*), and the Devonian green alga (*25*) show sizes overlapping with *Q. magnifica*, but their filaments have constant width and do not taper. In addition, similar filaments with diameter of 50 to 60 μm and long cylindrical cells were reported from the Beidajian Formation, Ruyang Group in southern NCC (*75*). They might represent the same species given their similar age to our fossils, which will need further investigation to confirm.

## MATERIALS AND METHODS

### Fossil preparation, extraction, and measurement

Twenty-three shale samples were processed to extract microfossils using palynological preparation technique at the Experimental Technologies Center of Nanjing Institute of Geology and Palaeontology, Chinese Academy of Sciences (NIGPAS). No oxidation was applied to organic residues. Tiny organic particles were removed by filtration using a nylon mesh with pore size of 15 μm. A small portion of obtained residues for each sample was mounted on microscope slides to make permanent strew mounts with glycerol jelly as mounting medium. The rest was used to pick individual microfossils by micropipette under a binocular stereo microscope.

Measurement of fossils and extant taxa was carried out on scaled photomicrographs using software “TPSDIGW32” (https://sbmorphometrics.org/soft-dataacq.html). For microfossils, specimens with no folds or only slightly folded were measured to obtain cell size data. Definitions of cell width and cell length are exemplified in fig. S2B.

### Transmitted light microscopy

Microfossils were examined in wet mounts and permanent strew mounts under a Nikon Eclipse Ni-U microscope and photographed under 20× and 40× objectives via an attached DS-Fi1c camera to the microscope using the software NIS-Elements F 4.00.00.

### Scanning electron microscopy

SEM analysis was conducted on TESCAN MAIA 3 GMU field-emission SEM with a secondary electron detector and operated at 10-keV accelerating voltage at the Experimental Technologies Center of NIGPAS. Before SEM examination, specimens were deposited on glass slide, left overnight for drying, and then coated with platinum for 60 s.

### Raman spectroscopy

Raman analysis was performed on isolated specimens deposited on glass sides using WITec 300R confocal Raman microscope at the Institute of Deep-sea Science and Engineering, Chinese Academy of Sciences (IDSSE). The instrument was calibrated using a standard silicon wafer at band position of 520.7 cm^−1^. The laser has 532 nm of wavelength and power of ~0.2 mW controlled by the True Power System. Spectra were acquired with laser focused through a 100× objective, 1 × 4-s running time, grating of 600 g/mm, and a spectral range of 60 to 4000 cm^−1^. Raman data were processed with software Project FIVE 5.1. All spectra were baseline corrected using the shape of size 300. Raman parameters, i.e., position (P), intensity (I), and FWHM of bands were acquired by filters with spatial average size of 1. Spectra were deconvoluted using the protocol proposed in (*34*). The fitted bands (subpeaks) were named with the suffix “function” following the literature (*38*) to distinguish from original peaks. For example, G function at ~1580 cm^−1^ is used here to differentiate from the general G peak at ~1600 cm^−1^ before deconvolution.

The thermal maturity of microfossils was evaluated by estimating peak-burial temperatures using Raman reflectance (RmcRo%) (*33*) and FWHM of D1 (at ~1350 cm^−1^) and D2 (at ~1620 cm^−1^) subpeaks (*34*). The Raman reflectance is used as an equivalent to vitrinite reflectance (vRo%) and defined using the separation of D and G bands as: RmcRo% = 0.0537 × (G − D) − 11.21, for mature to highly mature organic matter (*33*). Deconvolution of Raman spectra was not performed in this application. Temperatures estimated using FWHM of D1 and D2 subpeaks were performed deconvolution first following the protocol described in (*34*) and then calculated by equations: (i) T(°C) = −2.15 × (FWHM − D1) + 478, with suggested temperature range of 200° to 400°C; (ii) T(°C) = −6.78 × (FWHM − D2) + 535, with suggested temperature range of 50° to 200°C.

The PCA was performed using the first-order region (1000 to 1800 cm^−1^) of averaged Raman spectra of individual microfossils in the software Origin. Before analysis, 10 spectra with high signal-to-noise ratio were selected for each specimen. Spectra were baseline-corrected, truncated between 1000 and 1800 cm^−1^. Spectral intensity was normalized from 0 to 1 and then averaged 10 times for each specimen.

### FTIR spectroscopy

FTIR analysis was conducted on isolated microfossils deposited on potassium bromide (KBr) plates at room temperature using a Nicolet iS50 FTIR spectrometer coupled to a Continuμm microscope and equipped with liquid nitrogen cooled mercury cadmium telluride (MCT) detector at IDSSE. Spectra were acquired in transmission mode using 15× Reflachromat objective by accumulating 128 scans with an aperture of 50 μm by 50 μm in the range of 4000 to 650 cm^−1^. Spectral resolution is 4 cm^−1^. Baseline correction, truncation of spectra (4000 to 900 cm^−1^), and removal of CO_2_ peaks were processed in LabSpec 6 software.

## Supplementary Materials

### This PDF file includes:

Figs S1 to S6 Tables S1 to S4 Legend for table S5 Legends for data S1 to S3 References
