# Supplementary material for: 1.63-billion-year-old multicellular eukaryotes from the Chuanlinggou Formation in North China
Source: Sci Adv. 2024 Jan 24;10(4):eadk3208. doi: 10.1126/sciadv.adk3208 (PMC10807817; doi:10.1126/sciadv.adk3208)
Supplement: Supplementary file 1 — Figs S1 to S6 Tables S1 to S4 Legend for table S5 Legends for data S1 to S3 References [file sciadv.adk3208_sm.pdf]

Science Advances
AAAS

Supplementary Materials for

1.63-billion-year-old multicellular eukaryotes from the Chuanlinggou Formation in North China

Lanyun Miao *et al.*

Corresponding author: Maoyan Zhu, myzhu@nigpas.ac.cn

Sci. Adv. **10**, eadk3208 (2024)
DOI: 10.1126/sciadv.adk3208

The PDF file includes:

Figs. S1 to S6
Tables S1 to S4
Legend for table S5
Legends for data S1 to S3
References

Other Supplementary Material for this manuscript includes the following:

Table S5
Data S1 to S3

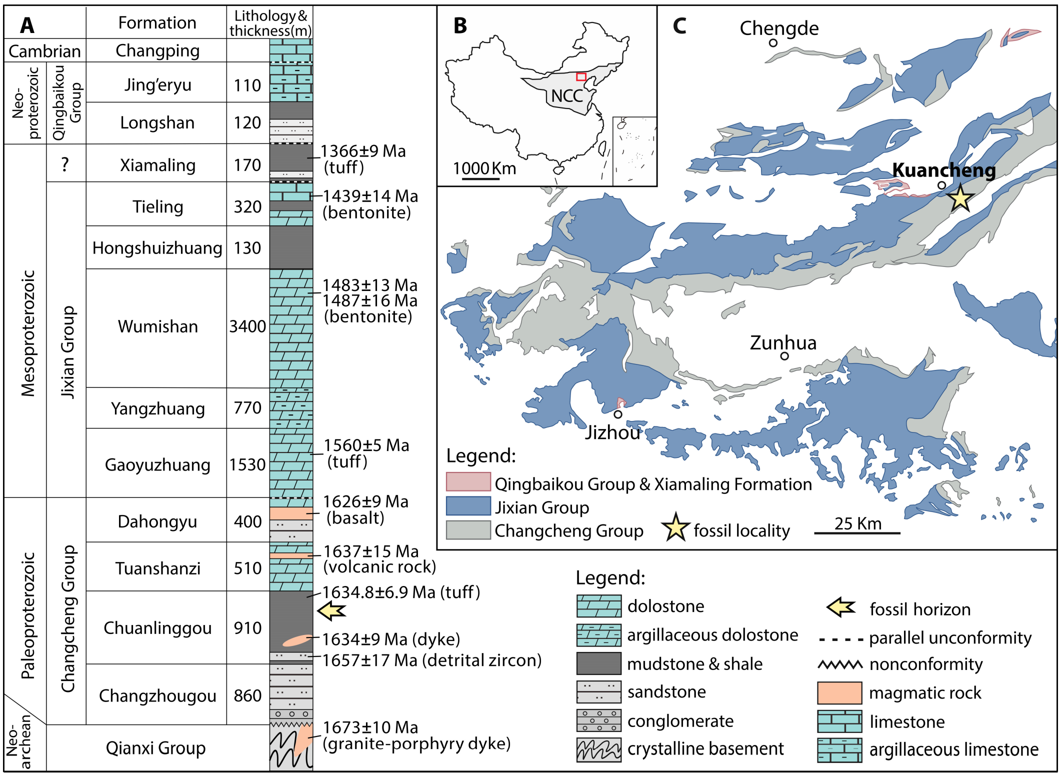

Fig. S1. Stratigraphy and geological map of the study area. (**A**) Generalized stratigraphic column showing Proterozoic strata in the Yanshan Range with age constraints and fossil horizon. Lithology is based on stratotype sections in Jizhou area from ref. (*80*). Radiometric dates are from refs. (*23, 81-87*). (**B**) Location map of the North China Craton (NCC). (**C**) A simplified geological map showing Proterozoic outcrops in study area (red box in B) with fossil locality.

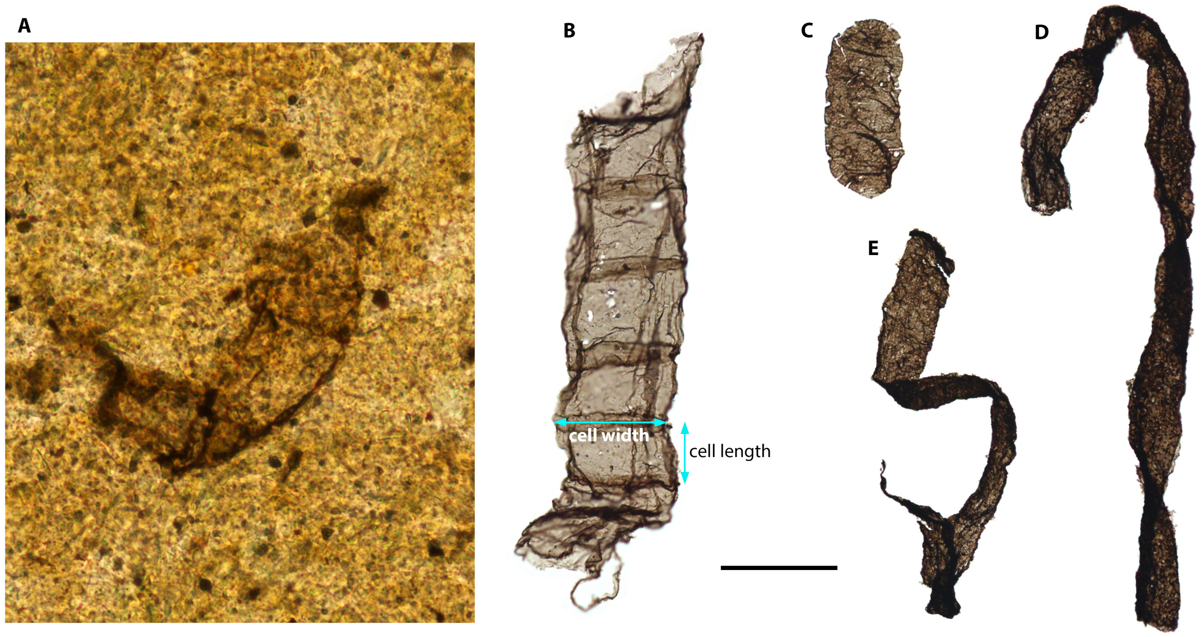

Fig. S2. **Transmitted-light (TL) photomicrographs of *Qingshania magnifica* and co-occurring taxa from the Chuanlinggou Formation**. (**A**, **B**) *Q. magnifica*. (**C**) *Oscillatoriopsis princeps*. (**D**) *Siphonophycus punctatum*. (**E**) *Pseudodendron* sp. Specimen in (**A**) is from petrographic thin section cut parallel to shale lamination; the rest were hand-picked from organic residues of acid maceration and photographed in wet mounts. Scale bar equals 50 µm for (**A**-**C**) and 100 µm for (**D**, **E**).

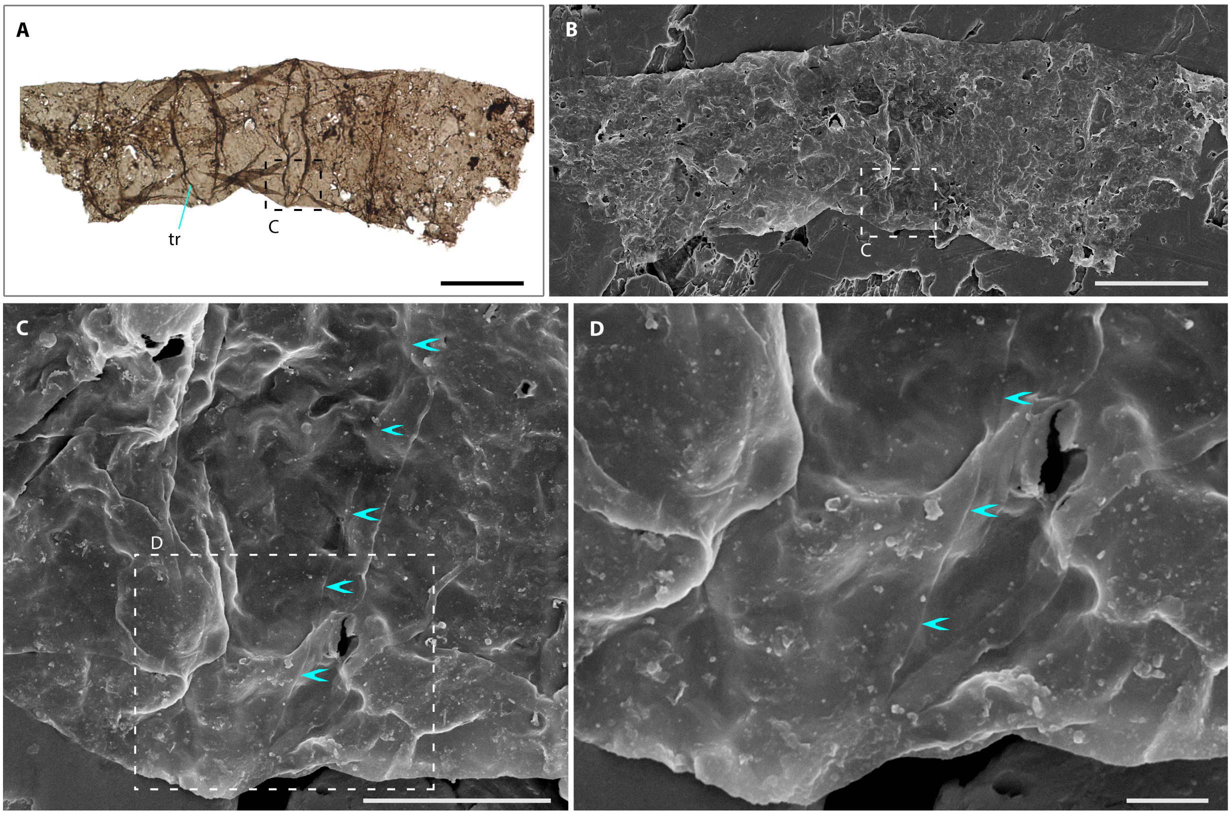

Fig. S3. Micrographs of *Qingshania magnifica* from the Chuanlinggou Formation. (**A**) TL photomicrograph of a filament with transverse rings (tr). (**B**) SEM image of (**A**). (**C**, **D**) Magnifications of (**B**), showing smooth wall surface and the well-defined contact between adjoining cells as indicated by a very shallow groove (marked by cyan arrowheads) along the transverse ring. (**C**) represents the dashed box in (**A**) and (**B**); (**D**) corresponds the dashed box in (**C**). tr: transverse ring (interpreted as partially preserved cross wall). Scale bars equal 50 µm for (**A**, **B**), 10 µm for (**C**), and 2 µm for (**D**).

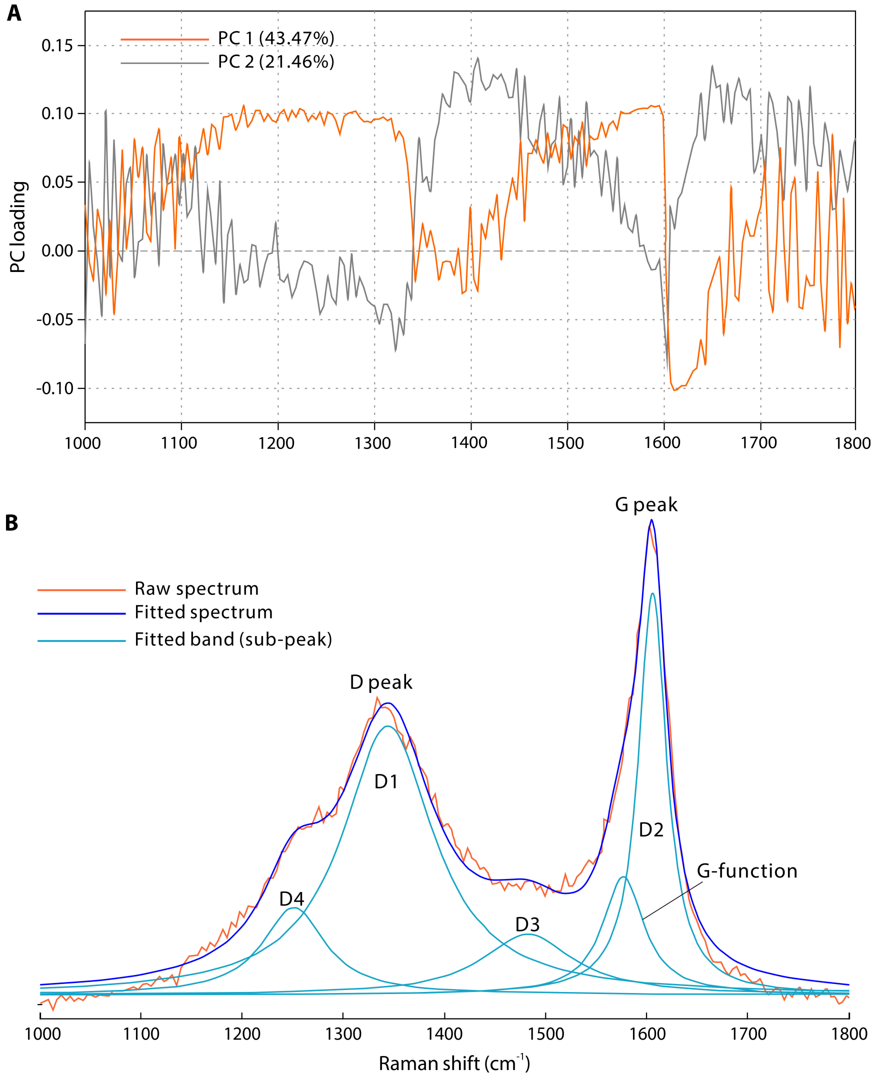

Fig. S4. Raman analyses of *Qingshania magnifica*. (**A**) PC loading plot. (**B**) Representative Raman spectrum showing fitted bands (sub-peaks). Spectrum was baselined corrected and decomposed following the peak fitting procedure in (*34*).

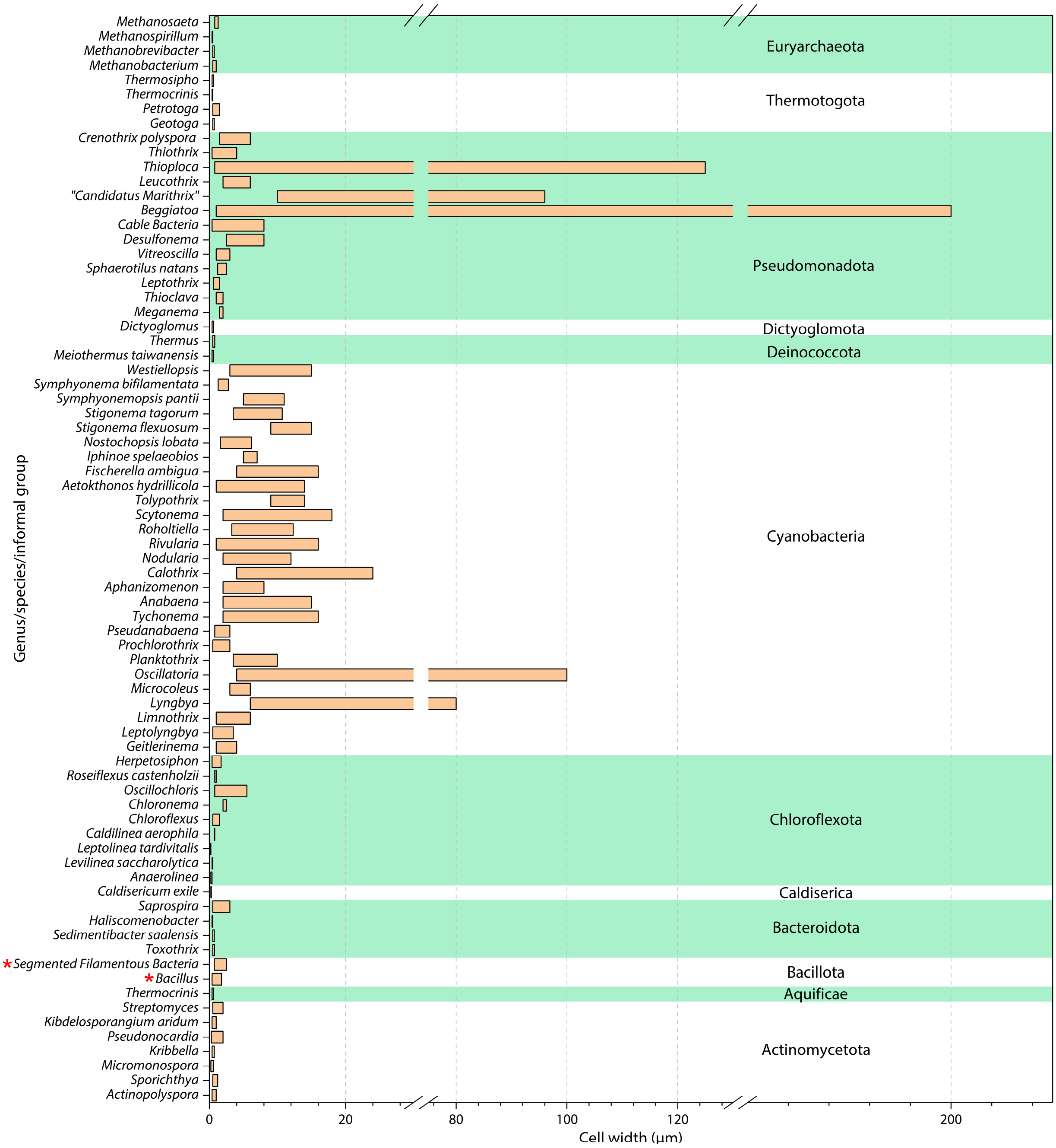

Fig. S5. Floating bar chart showing cell width range of extant uniseriate filamentous prokaryotes belonging to 11 bacterial and 1 archaeal phyla. Taxa producing endospore are marked with a red asterisk. Source data are provided in table S4 and cited references in table S5.

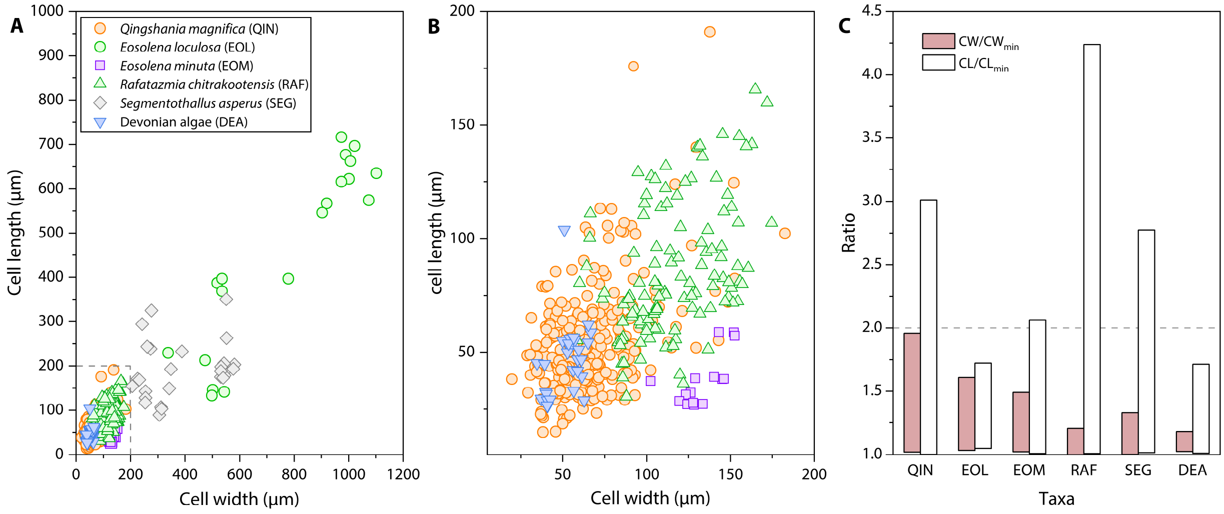

Fig. S6. Morphometric analyses of *Qingshania magnifica* and similar fossil taxa. (**A**, **B**) Scatter plots of cell length and cell width of filaments. (**B**) Magnification of the dashed box in (**A**). (**C**) Grouped floating bar chart showing ratios of cell width to minimum cell width (CW/CW_{min}) and cell length to minimum cell length (CL/CL_{min}) within single filaments. Abbreviations of taxa are provided in the legend of (**A**). Measurements of *Q. magnifica* are provided in Source Data 1. Size data of *Eosolena loculosa* (*9*), *Eosolena minuta* (*12*), *Rafatazmia chitrakootensis* (*14*), *Segmentothallus asperus* (*78*) and Devonian algae (*25*) were measured from scaled illustrations in the literature or directly cited from the literature and provided in Source Data 3.

Table S1. Measurement of selected extant eukaryotic algae and filamentous bacteria.

Chaetomorpha (*56*)					
Image measured	Cell no.	Cell width (μm)	Cell length (μm)	Width/width$_{min}$	Length/length$_{min}$
fig. 4	filament 1, cell 1	156.10	225.28	1.04	1.22
	filament 1, cell 2	161.31	236.23	1.08	1.28
	filament 1, cell 3	183.97	264.58	1.23	1.43
	filament 1, cell 4	173.25	184.69	1.16	1.00
	filament 1, cell 5	162.53	317.35	1.08	1.72
	filament 1, cell 6	149.84	281.80	1.00	1.53
	filament 2, cell 1	250.34	235.60	1.04	1.71
	filament 2, cell 2	241.21	218.81	1.00	1.59
	filament 2, cell 3	260.62	200.71	1.08	1.45
	filament 2, cell 4	271.60	184.69	1.13	1.34
	filament 2, cell 5	277.37	308.00	1.15	2.23
	filament 2, cell 6	260.87	138.03	1.08	1.00
	filament 3, cell 1	209.72	249.48	1.18	1.54
	filament 3, cell 2	185.31	244.67	1.04	1.51
	filament 3, cell 3	216.38	288.58	1.21	1.78
	filament 3, cell 4	222.03	164.55	1.25	1.02
	filament 3, cell 5	178.23	277.73	1.00	1.72
	filament 3, cell 6	185.67	336.73	1.04	2.08
	filament 3, cell 7	179.89	161.92	1.01	1.00
	filament 4, cell 1	471.40	408.11	1.02	2.60
	filament 4, cell 2	473.43	310.78	1.03	1.98
	filament 4, cell 3	465.59	304.77	1.01	1.94
	filament 4, cell 4	460.99	156.84	1.00	1.00
	filament 4, cell 5	460.60	218.43	1.00	1.39
	filament 5, cell 1	436.49	418.33	1.05	2.14
	filament 5, cell 2	427.89	228.99	1.03	1.17
	filament 5, cell 3	426.19	195.71	1.03	1.00
	filament 5, cell 4	415.68	364.24	1.00	1.86
	filament 5, cell 5	443.39	427.12	1.07	2.18
fig. 14	filament 1, cell 1	454.54	763.02	1.00	1.00
	filament 1, cell 2	529.18	1394.47	1.16	1.83
	filament 1, cell 3	555.43	1019.66	1.22	1.34
	filament 1, cell 4	632.77	1149.02	1.39	1.51
	filament 1, cell 5	710.72	984.78	1.56	1.29
	filament 1, cell 6	684.34	1099.48	1.51	1.44
	filament 2, cell 1	632.38	658.18	1.12	1.27
	filament 2, cell 2	645.66	916.47	1.14	1.78
	filament 2, cell 3	878.25	516.28	1.55	1.00
	filament 2, cell 4	903.58	801.65	1.59	1.55
	filament 2, cell 5	710.72	1111.53	1.25	2.15
	filament 2, cell 6	566.85	758.97	1.00	1.47
	filament 2, cell 7	910.55	676.02	1.61	1.31
	filament 3, cell 1	697.24	813.81	1.05	1.00
	filament 3, cell 2	839.59	980.71	1.26	1.21
	filament 3, cell 3	1045.86	929.10	1.57	1.14
	filament 3, cell 4	1071.64	981.39	1.61	1.21
	filament 3, cell 5	1007.17	864.88	1.52	1.06

	filament 3, cell 6	1046.46	1174.45	1.58	1.44
	filament 3, cell 7	683.98	1097.43	1.03	1.35
	filament 3, cell 8	664.22	827.50	1.00	1.02
fig. 6	filament 1, cell 1	244.31	246.75	1.06	1.00
	filament 1, cell 2	242.06	289.97	1.05	1.18
	filament 1, cell 3	242.06	465.79	1.05	1.89
	filament 1, cell 4	262.81	480.01	1.14	1.95
	filament 1, cell 5	248.96	310.55	1.08	1.26
	filament 1, cell 6	235.07	307.42	1.02	1.25
	filament 1, cell 7	231.49	253.11	1.00	1.03
	filament 2, cell 1	400.44	297.08	1.07	1.24
	filament 2, cell 2	390.82	537.64	1.04	2.24
	filament 2, cell 3	401.02	271.40	1.07	1.13
	filament 2, cell 4	374.68	290.60	1.00	1.21
	filament 2, cell 5	395.00	240.33	1.05	1.00
fig. 8	filament 1, cell 1	87.32	256.72	1.08	1.00
	filament 1, cell 2	98.13	300.25	1.22	1.17
	filament 1, cell 3	80.66	434.46	1.00	1.69
	filament 1, cell 4	85.52	531.16	1.06	2.07
	filament 2, cell 1	82.42	108.15	1.23	1.00
	filament 2, cell 2	67.10	290.96	1.00	2.69
	filament 2, cell 3	78.04	236.05	1.16	2.18
	filament 2, cell 4	67.10	220.31	1.00	2.04
fig. 21	filament 1, cell 1	96.47	338.36	1.77	2.53
	filament 1, cell 2	87.66	170.68	1.61	1.28
	filament 1, cell 3	86.87	144.88	1.60	1.08
	filament 1, cell 4	82.75	165.49	1.52	1.24
	filament 1, cell 5	80.23	133.83	1.48	1.00
	filament 1, cell 6	58.44	133.83	1.08	1.00
	filament 1, cell 7	54.35	167.14	1.00	1.25
	filament 2, cell 1	98.22	150.41	2.13	1.25
	filament 2, cell 2	83.57	137.36	1.81	1.14
	filament 2, cell 3	70.37	132.81	1.52	1.10
	filament 2, cell 4	66.24	149.27	1.43	1.24
	filament 2, cell 5	54.35	124.58	1.18	1.03
	filament 2, cell 6	46.20	120.47	1.00	1.00
Chaetomorpha (*88*)					
Image measured	Cell no.	Cell width (μm)	Cell length (μm)	Width/width$_{min}$	Length/length$_{min}$
fig. 2	filament 1, cell 1	48.39	196.73	1.03	1.17
	filament 1, cell 2	48.47	167.72	1.03	1.00
	filament 1, cell 3	47.00	198.34	1.00	1.18
fig. 6	filament 2, cell 1	47.51	63.20	1.00	1.66
	filament 2, cell 2	52.40	38.07	1.10	1.00
	filament 2, cell 3	48.66	51.78	1.02	1.36
fig. 8	filament 3, cell 1	28.54	90.07	1.00	1.03
	filament 3, cell 2	38.09	99.76	1.33	1.14
	filament 3, cell 3	39.00	87.81	1.37	1.00
Chaetomorpha (89)					
Image measured	Cell no.	Cell width (μm)	Cell length (μm)	Width/width$_{min}$	Length/length$_{min}$

fig. 6	filament 1, cell 1	12.84	40.50	1.03	1.00
	filament 1, cell 2	12.84	43.07	1.03	1.06
	filament 1, cell 3	12.47	45.24	1.00	1.12
	filament 1, cell 4	12.67	85.24	1.02	2.10
	filament 1, cell 5	15.23	76.59	1.22	1.89
	filament 1, cell 6	16.39	78.90	1.31	1.95
	filament 1, cell 7	17.10	104.50	1.37	2.58
	filament 1, cell 8	26.13	107.09	2.10	2.64
fig. 8	filament 1, cell 1	11.39	41.42	1.04	1.00
	filament 1, cell 2	10.93	65.66	1.00	1.59
	filament 1, cell 3	13.57	59.57	1.24	1.44
	filament 1, cell 4	14.42	54.63	1.32	1.32
	filament 1, cell 5	13.19	48.27	1.21	1.17
	filament 1, cell 6	17.49	73.71	1.60	1.78
	filament 2, cell 1	10.46	40.56	1.00	1.00
	filament 2, cell 2	12.03	46.76	1.15	1.15
	filament 2, cell 3	14.97	76.48	1.43	1.89
	filament 2, cell 4	14.09	65.88	1.35	1.62
	filament 2, cell 5	18.32	70.73	1.75	1.74
Chaetomorpha (*90*)					
Image measured	Cell no.	Cell width (µm)	Cell length (µm)	Width/width$_{min}$	Length/length$_{min}$
fig. 1	filament 1, cell 1	259.50	113.03	1.00	1.00
	filament 1, cell 2	270.77	180.60	1.04	1.60
	filament 1, cell 3	274.45	150.79	1.06	1.33
	filament 1, cell 4	295.62	254.66	1.14	2.25
	filament 1, cell 5	280.24	140.91	1.08	1.25
	filament 2, cell 1	159.49	147.81	1.01	1.64
	filament 2, cell 2	173.09	124.57	1.09	1.38
	filament 2, cell 3	161.69	90.30	1.02	1.00
	filament 2, cell 4	158.29	165.79	1.00	1.84
	filament 2, cell 5	169.84	200.13	1.07	2.22
	filament 3, cell 1	122.51	313.02	1.00	1.52
	filament 3, cell 2	136.73	205.53	1.12	1.00
	filament 3, cell 3	151.76	223.48	1.24	1.09
	filament 4, cell 1	104.56	122.77	1.04	1.00
	filament 4, cell 2	100.97	136.88	1.00	1.11
	filament 4, cell 3	104.38	194.37	1.04	1.58
	filament 4, cell 4	100.78	125.95	1.00	1.03
fig. 2	filament 1, cell 1	200.01	224.01	1.09	1.01
	filament 1, cell 2	200.97	305.64	1.10	1.38
	filament 1, cell 3	182.80	221.28	1.00	1.00
	filament 1, cell 4	199.25	425.41	1.09	1.92
	filament 2, cell 1	119.50	113.96	1.08	1.15
	filament 2, cell 2	114.30	113.96	1.03	1.15
	filament 2, cell 3	122.13	201.10	1.11	2.03
	filament 2, cell 4	113.41	98.88	1.03	1.00
	filament 2, cell 5	119.33	125.13	1.08	1.27
	filament 2, cell 6	110.50	192.06	1.00	1.94
	filament 2, cell 7	116.60	139.80	1.06	1.41
Urospora (*55*)					

Image measured	Cell no.	Cell width (µm)	Cell length (µm)	$Width/width_{min}$	$Length/length_{min}$
fig. 8A	filament 1, cell 1	32.84	80.98		
fig. 8B	filament 1, cell 1	43.47	55.07	1.01	1.00
fig. 8C	filament 1, cell 2	43.04	55.82	1.00	1.01
	filament 1, cell 3	86.18	131.10	2.00	2.38
fig. 9G	filament 1, cell 1	79.61	33.07	1.03	1.07
	filament 1, cell 2	77.29	30.78	1.00	1.00
	filament 1, cell 3	76.92	36.22	1.00	1.18
fig. 10A	filament 1, cell 1	147.03	88.33	1.00	1.00
	filament 1, cell 2	155.51	102.98	1.06	1.17
	filament 1, cell 3	189.24	233.84	1.29	2.65
	filament 1, cell 4	264.14	192.81	1.80	2.18
	filament 1, cell 5	257.00	194.59	1.75	2.20
fig. 10B	filament 1, cell 6	339.27	379.66	2.31	4.30
fig. 10C	filament 1, cell 7	330.15	282.02	2.25	3.19
	filament 1, cell 8	311.25	145.10	2.12	1.64
	filament 1, cell 9	322.01	122.64	2.19	1.39
fig. 11A	filament 1, cell 1	90.26	46.81	1.94	1.53
	filament 1, cell 2	95.59	34.32	2.05	1.12
	filament 1, cell 3	99.86	51.60	2.14	1.68
	filament 1, cell 4	96.66	61.09	2.08	1.99
	filament 1, cell 5	91.63	38.83	1.97	1.27
	filament 1, cell 6	90.03	43.46	1.93	1.42
	filament 1, cell 7	56.28	38.56	1.21	1.26
	filament 1, cell 8	59.42	45.07	1.28	1.47
	filament 1, cell 9	54.61	30.67	1.17	1.00
	filament 1, cell 10	46.58	32.15	1.00	1.05
	filament 1, cell 11	48.19	35.36	1.03	1.15
fig. 17B	filament 1, cell 1	87.62	47.94	1.10	1.53
	filament 1, cell 2	84.27	31.43	1.05	1.00
	filament 1, cell 3	122.96	62.97	1.54	2.00
	filament 1, cell 4	132.21	90.92	1.65	2.89
	filament 1, cell 5	138.93	95.95	1.74	3.05
	filament 1, cell 6	143.80	103.23	1.80	3.28
	filament 1, cell 7	79.89	48.25	1.00	1.54
	filament 1, cell 8	109.96	56.57	1.38	1.80
fig. 17F	filament 1, cell 1	56.52	38.77	1.05	1.55
	filament 1, cell 2	56.82	25.04	1.06	1.00
	filament 1, cell 3	58.03	28.95	1.08	1.16
	filament 1, cell 4	53.67	31.22	1.00	1.25
fig. 17G	filament 1, cell 1	28.62	35.46	1.00	1.00
	filament 1, cell 2	36.90	41.34	1.29	1.17
	filament 1, cell 3	32.70	40.13	1.14	1.13
	filament 1, cell 4	33.68	37.40	1.18	1.05
fig. 20G	filament 1, cell 1	55.72	40.99	1.00	1.00
	filament 1, cell 2	60.00	46.78	1.08	1.14
	filament 1, cell 3	60.43	49.24	1.08	1.20
Urospora (*46*)					
Image measured	Cell no.	Cell width (µm)	Cell length (µm)	$Width/width_{min}$	$Length/length_{min}$

fig. 18.11	filament 1, cell 1	53.70	124.21		
	filament 1, cell 2	55.14	110.63		
	filament 2, cell 1	57.14	105.37	1.00	1.18
	filament 2, cell 2	57.14	89.68	1.00	1.00
	filament 2, cell 3	58.39	89.48	1.02	1.00
	filament 3, cell 1	52.44	95.22	1.00	1.43
	filament 3, cell 2	53.22	92.13	1.01	1.38
	filament 3, cell 3	55.29	86.62	1.05	1.30
	filament 3, cell 4	55.87	66.66	1.07	1.00
Oscillatoria princeps (91)					
Image measured	Cell no.	Cell width (μm)	Cell length (μm)	Width/width$_{min}$	Length/length$_{min}$
fig. 4.32-4.33	filament 1, cell 1	37.72	7.26	1.03	1.61
	filament 1, cell 2	37.51	9.00	1.03	1.99
	filament 1, cell 3	37.24	8.77	1.02	1.94
	filament 1, cell 4	36.52	4.52	1.00	1.00
	filament 1, cell 5	37.30	6.78	1.02	1.50
	filament 1, cell 6	36.51	4.80	1.00	1.06
Oscillatoria princeps (*92*)					
Image measured	Cell no.	Cell width (μm)	Cell length (μm)	Width/width$_{min}$	Length/length$_{min}$
fig. 4	filament 1, cell 1	66.35	7.45	1.00	1.15
	filament 1, cell 2	67.58	7.48	1.02	1.16
	filament 1, cell 3	66.71	7.83	1.01	1.21
	filament 1, cell 4	66.22	6.46	1.00	1.00
	filament 1, cell 5	66.34	8.45	1.00	1.31
	filament 1, cell 6	66.46	8.32	1.00	1.29
fig. 11	filament 1, cell 1	62.04	4.75	1.01	1.00
	filament 1, cell 2	62.23	7.82	1.01	1.65
	filament 1, cell 3	62.21	7.29	1.01	1.53
	filament 1, cell 4	61.59	7.12	1.00	1.50
	filament 1, cell 5	61.78	8.26	1.00	1.74
	filament 2, cell 1	60.80	6.29	1.02	1.32
	filament 2, cell 2	60.32	5.24	1.01	1.10
	filament 2, cell 3	61.26	4.78	1.03	1.00
	filament 2, cell 4	60.19	6.56	1.01	1.37
	filament 2, cell 5	59.71	5.12	1.00	1.07
Oscillatoria princeps (*93*)					
Image measured	Cell no.	Cell width (μm)	Cell length (μm)	Width/width$_{min}$	Length/length$_{min}$
fig. 2C	filament 1, cell 1	105.50	25.45	1.05	1.30
	filament 1, cell 2	105.92	27.25	1.06	1.39
	filament 1, cell 3	103.29	22.21	1.03	1.13
	filament 1, cell 4	100.37	19.62	1.00	1.00
fig. 2J	filament 1, cell 1	26.20	5.34	1.09	1.00
	filament 1, cell 2	26.42	5.89	1.10	1.10
	filament 1, cell 3	25.37	6.13	1.05	1.15
	filament 1, cell 4	25.40	6.06	1.05	1.13
	filament 1, cell 5	24.09	5.59	1.00	1.05
	filament 2, cell 1	24.59	5.10	1.00	1.07
	filament 2, cell 2	24.97	6.01	1.02	1.26

	filament 2, cell 3	25.26	5.10	1.03	1.07
	filament 2, cell 4	25.67	4.78	1.04	1.00
Beggiatoa sp. (*51*)					
Image measured	Cell no.	Cell width (μm)	Cell length (μm)	Width/width$_{min}$	Length/length$_{min}$
fig. 3	filament 1, cell 1	12.19	4.16	1.05	1.13
	filament 1, cell 2	11.58	5.23	1.00	1.13
	filament 1, cell 3	12.06	5.74	1.04	1.56
	filament 1, cell 4	12.34	5.97	1.07	1.63
	filament 1, cell 5	12.17	4.89	1.05	1.33
	filament 1, cell 6	12.05	5.82	1.04	1.59
	filament 1, cell 7	11.98	5.08	1.03	1.38
	filament 1, cell 8	12.09	5.57	1.04	1.52
	filament 1, cell 9	11.81	4.13	1.02	1.13
	filament 1, cell 10	11.84	3.67	1.02	1.00
	filament 1, cell 11	12.02	4.60	1.04	1.25
	filament 1, cell 12	11.85	4.08	1.02	1.11
	filament 1, cell 13	12.02	5.94	1.04	1.62
	filament 1, cell 14	11.66	4.92	1.01	1.34
	filament 1, cell 15	11.95	5.21	1.03	1.42
	filament 1, cell 16	11.83	5.09	1.02	1.39
Beggiatoa spp. (*94*)					
Image measured	Cell no.	Cell width (μm)	Cell length (μm)	Width/width$_{min}$	Length/length$_{min}$
fig. 5A	filament 1, cell 1	40.97	10.94	1.02	1.07
	filament 1, cell 2	40.96	12.09	1.02	1.18
	filament 1, cell 3	41.53	10.94	1.03	1.07
	filament 1, cell 4	40.28	10.24	1.00	1.00
	filament 1, cell 5	41.96	12.09	1.04	1.18
	filament 1, cell 6	42.07	11.28	1.04	1.10
	filament 1, cell 7	40.63	11.28	1.01	1.10
	filament 1, cell 8	41.05	10.47	1.02	1.02
	filament 1, cell 9	41.15	10.89	1.02	1.06
	filament 2, cell 1	31.13	12.04	1.16	1.34
	filament 2, cell 2	28.80	9.27	1.08	1.03
	filament 2, cell 3	28.49	10.89	1.06	1.21
	filament 2, cell 4	28.74	10.89	1.07	1.21
	filament 2, cell 5	28.74	12.90	1.07	1.43
	filament 2, cell 6	28.52	11.20	1.07	1.25
	filament 2, cell 7	29.22	12.22	1.09	1.36
	filament 2, cell 8	26.77	12.60	1.00	1.40
	filament 2, cell 9	27.74	10.39	1.04	1.16
	filament 2, cell 10	28.76	10.60	1.07	1.18
	filament 2, cell 11	28.38	8.99	1.06	1.00
	filament 3, cell 1	39.47	8.43	1.06	1.00
	filament 3, cell 2	38.66	9.05	1.04	1.07
	filament 3, cell 3	37.36	8.49	1.00	1.01
	filament 3, cell 4	38.55	9.30	1.04	1.10
	filament 3, cell 5	37.18	8.74	1.00	1.04
	filament 3, cell 6	38.51	10.16	1.04	1.21
	filament 3, cell 7	37.66	11.53	1.01	1.37

fig. 5B	filament 4, cell 1	149.64	16.57	1.10	1.06
	filament 4, cell 2	149.71	15.70	1.10	1.00
	filament 4, cell 3	146.29	23.32	1.08	1.49
	filament 4, cell 4	144.55	22.47	1.06	1.43
	filament 4, cell 5	143.69	25.42	1.06	1.62
	filament 4, cell 6	136.02	27.55	1.00	1.75
	filament 4, cell 7	142.48	23.79	1.05	1.52
	filament 4, cell 8	142.98	23.40	1.05	1.49
Beggiatoa sp. (*95*)					
Image measured	Cell no.	Cell width (µm)	Cell length (µm)	Width/width$_{min}$	Length/length$_{min}$
fig. 2A	filament 1, cell 1	60.45	13.16	1.00	1.06
	filament 1, cell 2	61.54	12.47	1.02	1.00
	filament 1, cell 3	63.15	12.93	1.04	1.04
	filament 1, cell 4	62.56	17.34	1.03	1.39
	filament 1, cell 5	62.72	17.76	1.04	1.42
	filament 1, cell 6	61.40	14.96	1.02	1.20
	filament 2, cell 1	26.02	12.62	1.00	1.13
	filament 2, cell 2	26.47	11.73	1.02	1.05
	filament 2, cell 3	26.31	11.19	1.01	1.00
	filament 3, cell 1	23.28	12.83	1.00	1.08
	filament 3, cell 2	23.47	16.90	1.01	1.43
	filament 3, cell 3	24.18	11.85	1.04	1.00
	filament 3, cell 4	23.63	15.58	1.02	1.31
Beggiatoa sp. (*96*)					
Image measured	Cell no.	Cell width (µm)	Cell length (µm)	Width/width$_{min}$	Length/length$_{min}$
fig. 4	filament 1, cell 1	188.43	22.56	1.00	1.43
	filament 1, cell 2	190.53	26.44	1.01	1.67
	filament 1, cell 3	191.29	20.83	1.02	1.32
	filament 1, cell 4	191.18	20.08	1.01	1.27
	filament 1, cell 5	192.91	19.61	1.02	1.24
	filament 1, cell 6	191.23	22.19	1.01	1.40
	filament 1, cell 7	191.56	21.86	1.02	1.38
	filament 1, cell 8	192.37	21.46	1.02	1.36
	filament 1, cell 9	193.34	16.90	1.03	1.07
	filament 1, cell 10	194.74	16.94	1.03	1.07
	filament 1, cell 11	194.97	15.83	1.03	1.00
Beggiatoa sp. (*97*)					
Image measured	Cell no.	Cell width (µm)	Cell length (µm)	Width/width$_{min}$	Length/length$_{min}$
fig. 6D	filament 1, cell 1	6.40	2.51	1.00	1.00
	filament 1, cell 2	6.55	4.09	1.02	1.63
	filament 1, cell 3	6.71	3.94	1.05	1.57
	filament 1, cell 4	6.73	2.59	1.05	1.03
	filament 1, cell 5	6.62	2.70	1.03	1.08
	filament 1, cell 6	6.70	3.36	1.05	1.34
	filament 1, cell 7	6.80	3.05	1.06	1.22
	filament 1, cell 8	6.83	2.93	1.07	1.17
	filament 1, cell 9	6.86	2.81	1.07	1.12
	filament 1, cell 10	6.63	3.08	1.04	1.23

	filament 1, cell 11	6.98	3.05	1.09	1.22
	filament 1, cell 12	7.00	2.96	1.09	1.22
	filament 1, cell 13	6.92	2.87	1.08	1.18
Beggiatoa sp. (*98*)					
Image measured	Cell no.	Cell width (µm)	Cell length (µm)	Width/width$_{min}$	Length/length$_{min}$
fig. 1	filament 1, cell 1	41.84	19.35	1.03	1.00
	filament 1, cell 2	40.71	20.32	1.00	1.05
Beggiatoa sp. (*99*)					
Image measured	Cell no.	Cell width (µm)	Cell length (µm)	Width/width$_{min}$	Length/length$_{min}$
fig. 2a	filament 1, cell 1	56.72	15.17	1.00	1.00
	filament 1, cell 2	57.41	16.57	1.01	1.09
	filament 1, cell 3	57.31	17.29	1.01	1.14
fig. 2b	filament 2, cell 1	67.28	13.19	1.05	1.00
	filament 2, cell 2	63.82	14.38	1.00	1.09
	filament 2, cell 3	63.83	16.64	1.00	1.26
	filament 2, cell 4	64.85	18.71	1.02	1.42
fig. 2d	filament 3, cell 1	30.20	9.51	1.00	1.03
	filament 3, cell 2	30.31	9.23	1.00	1.00
	filament 3, cell 3	30.98	11.66	1.03	1.26
	filament 3, cell 4	30.28	10.43	1.00	1.13
	filament 3, cell 5	30.69	10.76	1.02	1.17
Thioploca spp. (*100*)					
Image measured	Cell no.	Cell width (µm)	Cell length (µm)	Width/width$_{min}$	Length/length$_{min}$
fig. 5	filament 1, cell 1	15.70	11.46	1.23	1.39
	filament 1, cell 2	15.43	9.34	1.21	1.13
	filament 1, cell 3	15.16	10.65	1.19	1.29
	filament 1, cell 4	14.90	13.04	1.17	1.58
	filament 1, cell 5	14.90	8.25	1.17	1.00
	filament 1, cell 6	12.78	10.67	1.00	1.29
	filament 2, cell 1	16.79	10.65	1.11	1.08
	filament 2, cell 2	16.77	10.12	1.11	1.03
	filament 2, cell 3	16.23	9.85	1.07	1.00
	filament 2, cell 4	15.43	11.71	1.02	1.19
	filament 2, cell 5	15.16	12.78	1.00	1.54
	filament 3, cell 1	38.67	20.85	1.00	1.00
	filament 3, cell 2	38.93	21.85	1.01	1.05
	filament 3, cell 3	38.86	21.80	1.00	1.05
	filament 3, cell 4	39.14	22.67	1.01	1.09
	filament 3, cell 5	38.94	21.24	1.01	1.02
	filament 4, cell 1	35.72	16.67	1.12	1.00
	filament 4, cell 2	36.27	18.01	1.14	1.08
	filament 4, cell 3	36.30	17.21	1.14	1.03
	filament 4, cell 4	32.81	20.15	1.03	1.21
	filament 4, cell 5	33.89	22.76	1.06	1.37
	filament 4, cell 6	31.93	23.40	1.00	1.40
Thioploca sp. (*101*)					
Image measured	Cell no.	Cell width (µm)	Cell length (µm)	Width/width$_{min}$	Length/length$_{min}$

Image measured	Cell no.	Cell width (μm)	Cell length (μm)	Width/width$_{min}$	Length/length$_{min}$
fig. 1d	filament 1, cell 1	45.80	46.09	1.04	1.00
	filament 1, cell 2	43.96	49.42	1.00	1.07
	filament 1, cell 3	44.38	56.86	1.01	1.23
	filament 1, cell 4	44.28	53.49	1.01	1.16
Thioploca sp. (*102*)					
Image measured	Cell no.	Cell width (μm)	Cell length (μm)	Width/width$_{min}$	Length/length$_{min}$
fig. 3	filament 1, cell 1	55.81	26.81	1.00	1.21
	filament 1, cell 2	56.16	22.19	1.01	1.00
	filament 1, cell 3	55.89	23.64	1.00	1.07
	filament 1, cell 4	56.47	23.22	1.01	1.05
	filament 1, cell 5	56.12	23.64	1.01	1.07
Thioploca spp. (*103*)					
Image measured	Cell no.	Cell width (μm)	Cell length (μm)	Width/width$_{min}$	Length/length$_{min}$
fig. 2B	filament 1, cell 1	39.51	24.50	1.04	1.04
	filament 1, cell 2	38.15	24.88	1.00	1.06
	filament 1, cell 3	39.42	25.91	1.03	1.10
	filament 1, cell 4	38.85	27.63	1.02	1.18
	filament 1, cell 5	43.05	23.47	1.13	1.00
	filament 2, cell 1	84.03	21.37	1.02	1.02
	filament 2, cell 2	82.02	29.77	1.00	1.41
	filament 2, cell 3	85.63	31.31	1.04	1.49
	filament 2, cell 4	87.00	22.30	1.06	1.06
	filament 2, cell 5	83.81	23.32	1.02	1.11
	filament 2, cell 6	84.56	21.05	1.03	1.00
	filament 2, cell 7	87.90	25.24	1.07	1.20

Table S2. FTIR band assignments. Abbreviations of taxa: QIN: *Qingshania magnifica*; SIP: *Siphonophycus punctatum*; PSEU: *Pseudodendron* sp.; OSCP: *Oscillatoriopsis princeps*. Estimation of relative band intensity: s = strong; m = medium; w = weak; "–" = absence of band; sh = band presence as shoulder. Band assignments are from (*40-42*).

Band wavenumber (cm^{-1})	Assignment	QIN	SIP	PSEU	OSCP
3400	hydroxyl O–H stretch	m	m	m	m
3050	aromatic =C–H stretch	w	w	w	w
2950	methyl CH_3 asymmetric stretch	sh	sh	sh	sh
2920	methylene CH_2 asymmetric stretch	w	w	w	w
2850	methylene CH_2 symmetric stretch	w	w	w	w
1735	carbonyl C=O stretch in ester	w, sh	–	–	–
1700	carbonyl C=O stretch in ketone, carboxylic acid, and aldehyde	w, sh	w, sh	w, sh	w, sh
1590	C=C aromatic ring stretch	s	s	s	s
1450	CH_2 and CH_3 deformation	m	m	s	–
1300	C–O stretch in carboxylic acid	m	–	–	–
1267	C–O stretch in aromatic ether, phenol, and carboxylic acid	m	m	m	m
1192	aromatic C–O asymmetric stretch	–	–	m	–
1125	aliphatic C–O–C asymmetric stretch	–	–	m	–
1080	aliphatic C–O stretch	–	–	m	–
1030	aromatic C–O stretch	sh	sh	sh	–

Table S3. Distribution of multicellularity in the Eukarya. “X” denotes the presence of the grade of organization. Lineages marked with an asterisk (*) represent photosynthetic organisms. The phylogeny of eukaryotes is from (*104, 105*).

Supergroup	Major group	Lineage	Unicell	Simple multicellularity	Complex multicellularity	Uniseriate filament
TSAR	Stramenopiles	Bicosoecids	X			
		Labyrinthulids	X			
		Thraustochytrids	X			
		Hyphochytrids	X	X		
		Actinophryids	X			
		Oomycetes	X	X		X
		*Diatoms	X	X		
		*Bolidophytes	X			
		*Phaeophytes		X	X	X
		*Dictyochophytes	X			
		*Pelagophytes	X	X		X
		*Chrysophytes	X	X		
		*Pinguiophytes	X			
		*Raphidophytes	X			
		*Synurophytes	X			
		*Xanthophytes	X	X		X
		*Eustigmatophytes	X			
	Alveolates	Colponemids	X			
		Ciliates	X			
		Acavamonas	X			
		Chrompodellids	X			
		Apicomplexans	X			
		Perkinsids	X			
		Oxyhrris	X			
		Syndinians	X			
		*Dinoflagellates	X			
	Rhizarians	Polycistenes	X			
		Acanthareans	X			
		Foraminiferans	X			
		Endomyxids	X			
		Monofilosids	X			
		Chlorarachniophytes	X			
		Mikrocytos	X			
		Haplosporidia	X			
		Gromia	X			
		Phytomyxea	X			
		Euglyphids	X			

		Cercomonads	X			
		Guttulinopsis		X		
		Reticulofilosids	X			
	Telonemids		X			
Haptista		*Haptophytes	X			
		*Rappemonads	X			
		Centrohelids	X			
		Ancoracysta	X			
		Microheliellids	X			
		Ancyromonads	X			
		Malawimonas	X			
		Hemimastigophorids	X			
		Picozoans	X			
Cryptista		Palpitomonas	X			
		*Cryptophytes	X			
		Katablepharids	X			
Archaeplastids		*Glaucophytes	X			
		Rhodelphis	X			
		*Red algae	X	X	X	X
		*Prasinophytes	X			
		*Trebouxiphytes	X	X		X
		*Chlorophytes	X	X		X
		*Charophytes	X	X		X
		*Tracheophytes			X	
Amorphea	Amoebozoans	Dictyostelids	X	X		
		Myxogastrids	acellular			
		Archamoebae	X			
		Varioseans	X			
		Cutoseans	X			
		Discoseans	X			
		Tubulinids	X	X		
		Breviates	X			
		Apusomonads	X			
	Opisthokonts	Ichthyosporeans		X		
		Filasterians	X	X		
		Choanoflagellates	X	X		
		Poriferans			X	
		Ctenophores			X	
		Cnidarians			X	
		Bilaterians			X	
		Nucleariids	X			
		Cryptomycetes	X			

		Microsporidians	X			
		Aphelids	X			
		Chytrids	X			
		Zygomycetes	X	X		X
		Dikaryans	X	X	X	X
CRuMS		Collodictyonids	X			
		Rigifilids	X			
		Mantamonads	X			
	Metamonads	Diplomonads	X			
		Parabasalids	X			
		Oxymonads	X			
	Discobids	Jakobids	X			
		Heteroloboseans	X	X		
		*Euglenids	X			
		Diplonemids	X			
		Kinetoplastids	X			

Table S4. Cell width of extant uniseriate filamentous prokaryotes.

Phylum	Order	Genus/species/informal group	Cell width (μm)	Reference
Actinomycetota	Actinomycetales	*Actinomyces*	0.2-1	(*106*)
Actinomycetota	Actinopolysporales	*Actinopolyspora*	0.4-1	(*107*)
Actinomycetota	Catenulisporales	*Catenulispora*	0.4-1.2 (spore)	(*108*)
Actinomycetota	Catenulisporales	*Actinospica*	0.6-1.2 (spore)	(*109*)
Actinomycetota	Corynebacteriales	*Rhodococcus jostii*	0.4-0.6	(*110*)
Actinomycetota	Frankiales	*Frankia*	0.5-2	(*111*)
Actinomycetota	Frankiales	*Acidothermus*	~ 0.4	(*112*)
Actinomycetota	Frankiales	*Geodermatophilus obscurus*	0.5-2	(*113*)
Actinomycetota	Frankiales	*Sporichthya*	0.5-1.2	(*114*)
Actinomycetota	Glycomycetales	*Glycomyces*	0.35-0.4	(*115*)
Actinomycetota	Glycomycetales	*Stackebrandtia*	~ 0.5	(*116*)
Actinomycetota	Jiangellales	*Jangella muralis*	~ 1.3	(*117*)
Actinomycetota	Jiangellales	*Haloactinopolyspora alba*	~ 0.4	(*118*)
Actinomycetota	Micrococcales	*Oerskovia*	~ 0.5	(*119*)
Actinomycetota	Micrococcales	*Tropheryma*	0.2-0.25	(*120*)
Actinomycetota	Micrococcales	*Dermatophilus*	0.5-1.5	(*121*)
Actinomycetota	Micrococcales	*Intrasporangium*	0.4-1.2	(*122*)
Actinomycetota	Micrococcales	*Humihabitans*	~ 0.5	(*123*)
Actinomycetota	Micrococcales	*Agromyces*	0.2-0.6	(*124*)
Actinomycetota	Micrococcales	*Leifeonia*	0.3-0.6	(*125*)
Actinomycetota	Micrococcales	*Promicromonospora*	0.5-1	(*126*)
Actinomycetota	Micrococcales	*Cellulosimicrobium terreum*	0.4-0.8	(*127*)
Actinomycetota	Micrococcales	*Isoptericola dokdonensis*	0.8-1.1	(*128*)
Actinomycetota	Micrococcales	*Mycellgenerans crystallogenes*	0.5-0.7	(*129*)
Actinomycetota	Micromonosporales	*Micromonospora*	0.2-0.6	(*130*)
Actinomycetota	Micromonosporales	*Actinocatenispora*	0.3-0.4 (spore)	(*131*)
Actinomycetota	Micromonosporales	*Actinoplanes*	0.2-1.2	(*132*)
Actinomycetota	Micromonosporales	*Asanoa*	0.3-0.4	(*133*)
Actinomycetota	Micromonosporales	*Catenuloplanes*	0.6-0.8 (spore)	(*134*)
Actinomycetota	Micromonosporales	*Couchioplanes*	0.5-0.9 (spore)	(*135*)
Actinomycetota	Micromonosporales	*Dactylosporangium*	0.5-1	(*136*)
Actinomycetota	Micromonosporales	*Krasilnikovia*	0.2-0.4 (spore)	(*137*)
Actinomycetota	Micromonosporales	*Longispora*	0.4-0.5 (spore)	(*138*)
Actinomycetota	Micromonosporales	*Luedemannella*	0.2-0.4 (spore)	(*139*)
Actinomycetota	Micromonosporales	*Pilimelia*	0.2-0.8	(*140*)
Actinomycetota	Micromonosporales	*Polymorphospora*	0.6-0.9 (spore)	(*141*)
Actinomycetota	Micromonosporales	*Salinispora*	0.25-0.5	(*142*)
Actinomycetota	Micromonosporales	*Spirilliplanes*	0.5-0.7 (spore)	(*143*)
Actinomycetota	Micromonosporales	*Verrucosispora*	0.4	(*144*)
Actinomycetota	Micromonosporales	*Virgisporangium*	0.6-0.9 (spore)	(*145*)
Actinomycetota	Propionibacteriales	*Kribbella*	0.4-0.7	(*146*)

Actinomycetota	Pseudonocardiales	*Pseudonocardia*	0.3-2	(*147*)
Actinomycetota	Pseudonocardiales	*Actinoalloteichus hymeniacidonis*	0.6-0.8 (spore)	(*148*)
Actinomycetota	Pseudonocardiales	*Actinokineospora*	~ 0.5	(*149*)
Actinomycetota	Pseudonocardiales	*Actinosynnema*	0.5-1	(*150*)
Actinomycetota	Pseudonocardiales	*Amycolatopsis nigrescens*	0.7-0.9	(*151*)
Actinomycetota	Pseudonocardiales	*Crossiella*	~ 0.5	(*152*)
Actinomycetota	Pseudonocardiales	*Goodfellowiella*	~ 0.5	(*153*)
Actinomycetota	Pseudonocardiales	*Kibdelosporangium aridum*	0.4-1	(*154*)
Actinomycetota	Pseudonocardiales	*Lechevalieria*	~ 0.5	(*155*)
Actinomycetota	Pseudonocardiales	*Lentzea*	0.5-0.7	(*156*)
Actinomycetota	Pseudonocardiales	*Prauserella*	0.6-0.8	(*157*)
Actinomycetota	Pseudonocardiales	*Saccharomonospora cyanea*	0.8-1 (spore)	(*158*)
Actinomycetota	Pseudonocardiales	*Saccharopolyspora hirsuta*	0.4-0.6	(*159*)
Actinomycetota	Pseudonocardiales	*Saccharothrix*	0.5-0.7	(*160*)
Actinomycetota	Pseudonocardiales	*Streptoalloteichus hindustanus*	~ 0.5	(*161*)
Actinomycetota	Pseudonocardiales	*Umezawaea*	0.3-0.5	(*162*)
Actinomycetota	Pseudonocardiales	*Streptosporangium fragile*	0.5-1	(*163*)
Actinomycetota	Pseudonocardiales	*Acrocarpospora*	0.6-0.8 (spore)	(*164*)
Actinomycetota	Pseudonocardiales	*Microbispora*	1-1.4 (spore)	(*165*)
Actinomycetota	Pseudonocardiales	*Microtetraspora*	1-1.5 (spore)	(*166*)
Actinomycetota	Pseudonocardiales	*Planobispora*	0.5-1	(*167*)
Actinomycetota	Pseudonocardiales	*Planomonospora*	0.6-1	(*168*)
Actinomycetota	Pseudonocardiales	*Planotetraspora*	0.4-1.4 (spore)	(*169*)
Actinomycetota	Pseudonocardiales	*Sphaerisporangium*	0.4-0.9 (spore)	(*170*)
Actinomycetota	Pseudonocardiales	*Thermopolyspora*	1.2-1.5 (spore)	(*171*)
Actinomycetota	Pseudonocardiales	*Nocardiopsis ganjiahuensis*	0.3-0.5 (spore)	(*172*)
Actinomycetota	Pseudonocardiales	*Haloactinospora*	0.4-0.6 (spore)	(*173*)
Actinomycetota	Pseudonocardiales	*Streptomonospora*	0.5-0.8	(*174*)
Actinomycetota	Pseudonocardiales	*Thermobifida*	0.5-2 (spore)	(*175*)
Actinomycetota	Pseudonocardiales	*Actinomadura atramentaria*	0.6-0.8 (spore)	(*176*)
Actinomycetota	Pseudonocardiales	*Spirillospora*	0.5-0.7 (spore)	(*177*)
Actinomycetota	Pseudonocardiales	*Thermobispora*	0.5-0.8	(*178*)
Actinomycetota	Streptomycetales	*Streptomyces*	0.5-2	(*179*)
Aquificae	Aquificales	*Thermocrinis*	0.4-0.6	(*180-182*)
Bacillota	Bacillales	*Bacillus*	0.4-1.8	(*183*)
Bacillota	Clostridiales	Segmented Filamentous Bacteria	0.7-2.5	(*53, 184*)
Bacteroidota	*Incertae sedis*	*Toxothrix*	0.5-0.75	(*185*)
Bacteroidota	Flavobacteriales	*Sedimentibacter saalensis*	0.5-0.7	(*186*)
Bacteroidota	Sphingobacteriales	*Haliscomenobacter*	0.4-0.5	(*187*)
Bacteroidota	Sphingobacteriales	*Saprospira*	0.5-3	(*188*)
Caldiserica	Caldisericales	*Caldisericum exile*	~ 0.3	(*189*)

Chloroflexota	Anaerolineales	*Anaerolinea*	0.2-0.4	(*190*)
Chloroflexota	Anaerolineales	*Levilinea saccharolytica*	0.4-0.5	(*190*)
Chloroflexota	Anaerolineales	*Leptolinea tardivitalis*	0.15-0.2	(*190*)
Chloroflexota	Caldilineales	*Caldilinea aerophila*	0.7-0.8	(*190*)
Chloroflexota	Chloroflexales	*Chloroflexus*	0.5-1.5	(*191*)
Chloroflexota	Chloroflexales	*Chloronema*	2-2.5	(*192*)
Chloroflexota	Chloroflexales	*Heliothrix*	~ 1.5	(*193*)
Chloroflexota	Chloroflexales	*Oscillochloris*	0.8-5.5	(*194*)
Chloroflexota	Chloroflexales	*Roseiflexus castenholzii*	0.8-1	(*195*)
Chloroflexota	Herpetosiphonales	*Herpetosiphon*	0.5-1.5	(*196-200*)
Cyanobacteria	Subsection III	*Geitlerinema*	1-4	(*201*)
Cyanobacteria	Subsection III	*Leptolyngbya*	0.5-3.5	(*202, 203*)
Cyanobacteria	Subsection III	*Limnothrix*	1-6	(*204, 205*)
Cyanobacteria	Subsection III	*Lyngbya*	6-80	(*50*)
Cyanobacteria	Subsection III	*Microcoleus*	3-6	(*206*)
Cyanobacteria	Subsection III	*Oscillatoria*	4-100	(*47*)
Cyanobacteria	Subsection III	*Planktothrix*	3.5-10	(*207*)
Cyanobacteria	Subsection III	*Prochlorothrix*	0.5-3	(*208, 209*)
Cyanobacteria	Subsection III	*Pseudanabaena*	0.8-3	(*210*)
Cyanobacteria	Subsection III	*Tychonema*	2-16	(*211*)
Cyanobacteria	Subsection IV	*Anabaena*	2-15	(*212*)
Cyanobacteria	Subsection IV	*Aphanizomenon*	2-8	(*213, 214*)
Cyanobacteria	Subsection IV	*Calothrix*	4-24	(*215*)
Cyanobacteria	Subsection IV	*Cuspidothrix*	< 6	(*214*)
Cyanobacteria	Subsection IV	*Cylindrospermum*	< 6	(*216, 217*)
Cyanobacteria	Subsection IV	*Nodularia*	2-12	(*218-221*)
Cyanobacteria	Subsection IV	*Nostoc*	< 12	(*222, 223*)
Cyanobacteria	Subsection IV	*Raphidiopsis*	< 4	(*224*)
Cyanobacteria	Subsection IV	*Rivularia*	1-16	(*48*)
Cyanobacteria	Subsection IV	*Roholtiella*	3.3-12.3	(*225*)
Cyanobacteria	Subsection IV	*Scytonema*	2-18	(*226, 227*)
Cyanobacteria	Subsection IV	*Tolypothrix*	9-14	(*228, 229*)
Cyanobacteria	Subsection V	*Aetokthonos hydrillicola*	1-14	(*230*)
Cyanobacteria	Subsection V	*Fischerella ambigua*	4-16	(*49*)
Cyanobacteria	Subsection V	*Fischerella thermalis*	4-8	(*231*)
Cyanobacteria	Subsection V	*Iphinoe spelaeobios*	5-7	(*232*)
Cyanobacteria	Subsection V	*Nostochopsis lobata*	1.6-6.2	(*233*)
Cyanobacteria	Subsection V	*Nostochopsis radians*	< 8.4	(*234*)
Cyanobacteria	Subsection V	*Stigonema crassivaginatum*	12-14	(*235*)
Cyanobacteria	Subsection V	*Stigonema flexuosum*	9-15	(*235*)
Cyanobacteria	Subsection V	*Stigonema cf. minutum*	6.5-10	(*235*)
Cyanobacteria	Subsection V	*Stigonema tagorum*	3.5-10.7	(*236*)

Cyanobacteria	Subsection V	*Stigonema tomentosum*	4-10	(*235*)
Cyanobacteria	Subsection V	*Symphyonemopsis pantii*	5-11	(*237*)
Cyanobacteria	Subsection V	*Symphyonema bifilamentata*	1.3-2.8	(*238*)
Cyanobacteria	Subsection V	*Westiellopsis*	3-15	(*239*)
Deinococcota	Thermales	*Meiothermus taiwanensis*	0.4-0.6	(*240*)
Deinococcota	Thermales	*Thermus*	0.5-0.8	(*241*)
Dictyoglomota	Dictyoglomales	*Dictyoglomus*	0.4-0.6	(*242*)
Pseudomonadota	Rhizobiales	*Meganema*	1.5-2	(*243*)
Pseudomonadota	Rhodobacterales	*Thioclava*	1-2	(*244*)
Pseudomonadota	Burkholderiales	*Leptothrix*	0.6-1.5	(*245, 246*)
Pseudomonadota	Burkholderiales	*Sphaerotilus natans*	1.2-2.5	(*246, 247*)
Pseudomonadota	Neisseriales	*Vitreoscilla*	1-3	(*248*)
Pseudomonadota	Desulfobacterales	*Desulfonema*	2.5-8	(*249*)
Pseudomonadota	Desulfobacterales	cable bacteria	0.4-8	(*250*)
Pseudomonadota	Thiotrichales	*Beggiatoa*	1-200	(*51*)
Pseudomonadota	Thiotrichales	"*Candidatus* Marithrix"	10-96	(*251*)
Pseudomonadota	Thiotrichales	*Leucothrix*	2-6	(*252, 253*)
Pseudomonadota	Thiotrichales	*Thioploca*	0.8-43, up to 125	(*52, 254*)
Pseudomonadota	Thiotrichales	*Thiothrix*	0.5-8	(*255-259*)
Pseudomonadota	Methylococcales	*Crenothrix polyspora*	1.5-6	(*260-262*)
Thermotogota	Thermotogales	*Geotoga*	0.5-0.7	(*263*)
Thermotogota	Thermotogales	*Petrotoga*	0.5-1.5	(*264*)
Thermotogota	Thermotogales	*Thermocrinis*	0.4-0.5	(*180*)
Thermotogota	Thermotogales	*Thermosipho*	0.4-0.6	(*265*)
Euryarchaeota	Methanobacteriales	*Methanobacterium*	0.5-1	(*266*)
Euryarchaeota	Methanobacteriales	*Methanobrevibacter*	0.5-0.7	(*267*)
Euryarchaeota	Methanobacteriales	*Methanoline tarda*	0.7-1	(*268*)
Euryarchaeota	Methanothermaceae	*Methanothermus*	0.3-0.4	(*269*)
Euryarchaeota	Methanomicrobiales	*Methanospirillum*	0.4-0.5	(*270*)
Euryarchaeota	Methanomicrobiales	*Methanosaeta*	0.8-1.3	(*271*)

Title for supplemental auxiliary files:

Table S5. References (106-271) cited in Table S4.

Data S1. Measurement and size frequency of *Qingshania magnifica*.

Data S2. Raman and FTIR data of *Qingshania magnifica*.

Data S3. Measurement of other fossil taxa.

REFERENCES AND NOTES

1. E. Szathmáry, Toward major evolutionary transitions theory 2.0. *Proc Natl Acad Sci U.S.A.* **112**, 10104–10111 (2015).

2. A. H. Knoll, The multiple origins of complex multicellularity. *Annu. Rev. Earth Planet. Sci.* **39**, 217–239 (2011).

3. N. J. Butterfield, Modes of pre-Ediacaran multicellularity. *Precambrian Res.* **173**, 201–211 (2009).

4. J. W. Schopf, A. B. Kudryavtsev, A. D. Czaja, A. B. Tripathi, Evidence of Archean life: Stromatolites and microfossils. *Precambrian Res.* **158**, 141–155 (2007).

5. N. J. Butterfield, *Bangiomorpha* pubescensn. gen., n. sp.: Implications for the evolution of sex, multicellularity, and the Mesoproterozoic/Neoproterozoic radiation of eukaryotes. *Paleobiology* **26**, 386–404 (2000).

6. T. M. Gibson, P. M. Shih, V. M. Cumming, W. W. Fischer, P. W. Crockford, M. S. W. Hodgskiss, S. Wörndle, R. A. Creaser, R. H. Rainbird, T. M. Skulski, G. P. Halverson, Precise age of *Bangiomorpha pubescens* dates the origin of eukaryotic photosynthesis. *Geology* **46**, 135–138 (2018).

7. Q. Tang, K. Pang, X. Yuan, S. Xiao, A one-billion-year-old multicellular chlorophyte. *Nat. Ecol. Evol.* **4**, 543–549 (2020).

8. C. C. Loron, C. François, R. H. Rainbird, E. C. Turner, S. Borensztajn, E. J. Javaux, Early fungi from the Proterozoic era in Arctic Canada. *Nature* **570**, 232–235 (2019).

9. T. N. German, V. N. Podkovyrov, New insights into the nature of the Late Riphean Eosolenides. *Precambrian Res.* **173**, 154–162 (2009).

10. M. C. Sforna, C. C. Loron, C. F. Demoulin, C. François, Y. Cornet, Y. J. Lara, D. Grolimund, D. Ferreira Sanchez, K. Medjoubi, A. Somogyi, A. Addad, A. Fadel, P. Compère, D. Baudet, J. J. Brocks, E. J. Javaux, Intracellular bound chlorophyll residues identify 1 Gyr-old fossils as eukaryotic *algae. Nat. Commun.* **13**, 146 (2022).

11. K. M. Maloney, D. P. Maverick, J. D. Schiffbauer, G. P. Halverson, S. Xiao, M. Laflamme, Systematic paleontology of macroalgal fossils from the Tonian Mackenzie Mountains Supergroup. *J. Paleo.* **97**, 499–515 (2023).

12. N. G. Vorob'eva, V. N. Sergeev, P. Y. Petrov, Kotuikan Formation assemblage: A diverse organic-walled microbiota in the Mesoproterozoic Anabar succession, northern Siberia. *Precambrian Res.* **256**, 201–222 (2015).

13. S. Zhu, M. Zhu, A. H. Knoll, Z. Yin, F. Zhao, S. Sun, Y. Qu, M. Shi, H. Liu, Decimetre-scale multicellular eukaryotes from the 1.56-billion-year-old Gaoyuzhuang Formation in North China. *Nat. Commun.* **7**, 11500 (2016).

14. S. Bengtson, T. Sallstedt, V. Belivanova, M. Whitehouse, Three-dimensional preservation of cellular and subcellular structures suggests 1.6 billion-year-old crown-group red algae. *PLOS Biol.* **15**, e2000735 (2017).

15. M. Sharma, Y. Shukla, Taxonomy and affinity of Early Mesoproterozoic megascopic helically coiled and related fossils from the Rohtas Formation, the Vindhyan Supergroup, India. *Precambrian Res.* **173**, 105–122 (2009).

16. M. R. Walter, R. Du, R. J. Horodyski, Coiled carbonaceous megafossils from the Middle Proterozoic of Jixian (Tianjin) and Montana. *Am. J. Sci.* **290**, 133–148 (1990).

17. T.-M. Han, B. Runnegar, Megascopic eukaryotic algae from the 2.1-billion-year-old Negaunee iron-formation, Michigan. *Science* **257**, 232–235 (1992).

18. A. E. Albani, S. Bengtson, D. E. Canfield, A. Bekker, R. Macchiarelli, A. Mazurier, E. U. Hammarlund, P. Boulvais, J.-J. Dupuy, C. Fontaine, F. T. Fürsich, F. Gauthier-Lafaye, P. Janvier, E. Javaux, F. O. Ossa, A.-C. Pierson-Wickmann, A. Riboulleau, P. Sardini, D. Vachard, M. Whitehouse, A. Meunier, Large colonial organisms with coordinated growth in oxygenated environments 2.1 Gyr ago. *Nature* **466**, 100–104 (2010).

19. S. Zhu, H. Chen, Megascopic multicellular organisms from the 1700-million-year-old Tuanshanzi formation in the Jixian area, North China. *Science* **270**, 620–622 (1995).

20. Y. Yan, Shale-facies algal filaments from Chuanlinggou Formation in Jixian County. *Bulletin of the Tianjin Institute of Geology and Mineral Resources* **21**, 149–165 (1989).

21. Z. Xu, B. Cui, Sinian Suberathem in the eastern Yanshan Ranges in *Research on Precambrian Geology - Sinian Suberathem in China*, C. G. S. Tianjin Institute of Geology and Mineral Resources, Ed. (Tianjin Science and Technology Press, 1980), pp. 358–369.

22. M. Jiang, J. Shi, T. Fan, W. Zhao, Z. Wu, J. Fan, Lithofacies and depositional settings of Mesoproterozoic shales: Insights from the ~1.65 Ga Chuanlinggou Formation, Yanliao Basin, North China Craton. *Geoenergy Science and Engineering* **229**, 211998 (2023).

23. D. Liu, X. Wang, H. Zhang, C. Shi, Zircon SHRIMP U-Pb age of the Chuanlinggou Formation of the Changcheng Group, North China and the stratigraphic implications. *Earth Sci. Front.* **26**, 183–189 (2019).

24. M. Moczydłowska, Algal affinities of Ediacaran and Cambrian organic-walled microfossils with internal reproductive bodies:Tanariumand other morphotypes. *Palynology* **40**, 83–121 (2016).

25. P. Filipiak, L. E. Graham, Z. Wawrzyniak, M. Kondas, Filamentous eukaryotic algae from the Lower Devonian, Bukowa Góra (Holy Cross Mountains, Poland). *Rev. Palaeobot. Palynol.* **288**, 104411 (2021).

26. B. Bomfleur, S. McLoughlin, V. Vajda, Fossilized nuclei and chromosomes reveal 180 million years of genomic stasis in royal ferns. *Science* **343**, 1376–1377 (2014).

27. F. Míguez, A. Holzinger, B. Fernandez-Marin, J. I. García-Plazaola, U. Karsten, L. Gustavs, Ecophysiological changes and spore formation: Two strategies in response to low-temperature and high-light stress in *Klebsormidium* Cf. *flaccidum* (Klebsormidiophyceae, Streptophyta)1. *J. Phycol.* **56**, 649–661 (2020).

28. G. M. Lokhorst, The genus *Tribonema* (Xanthophyceae) in the Netherlands An integrated field and culture study. *Nova Hedwigia Band* **77**, 19–53 (2003).

29. A. K. Mitra, On the structure and reproduction of *Uronema terrestre* n.sp. *Ann. Bot.* **11**, 349–361 (1947).

30. R. Giorno, J. Bozue, C. Cote, T. Wenzel, K.-S. Moody, M. Mallozzi, M. Ryan, R. Wang, R. Zielke, J. R. Maddock, A. Friedlander, S. Welkos, A. Driks, Morphogenesis of the *Bacillus anthracis* Spore. *J. Bacteriol.* **189**, 691–705 (2007).

31. V. G. Delvecchio, R. Corbaz, G. Turian, An ultrastructural study of the hyphae, endoconidia and chlamydospores of *Thielaviopsis basicola*. *J. Gen. Microbiol.* **58**, 23–27 (1969).

32. G. Lokhorst, B. Trask, Taxonomic studies on *Urospora* (Acrosiphoniales, Chlorophyceae) in western Europe. *Acta Botanica Neerlandica* **30**, 353–431 (1981).

33. D. Liu, X. Xiao, H. Tian, Y. Min, Q. Zhou, P. Cheng, J. Shen, Sample maturation calculated using Raman spectroscopic parameters for solid organics: Methodology and geological applications. *Chin. Sci. Bull.* **58**, 1285–1298 (2013).

34. Y. Kouketsu, T. Mizukami, H. Mori, S. Endo, M. Aoya, H. Hara, D. Nakamura, S. Wallis, A new approach to develop the Raman carbonaceous material geothermometer for low-grade metamorphism using peak width. *Island Arc* **23**, 33–50 (2014).

35. Y. Qu, A. Engdahl, S. Zhu, V. Vajda, N. McLoughlin, Ultrastructural heterogeneity of carbonaceous material in ancient cherts: Investigating biosignature origin and preservation. *Astrobiology* **15**, 825–842 (2015).

36. C. Beny-Bassez, J. N. Rouzaud, Characterization of carbonaceous materials by correlated electron and optical microscopy and Raman microspectroscopy. *Scan. Electron Microsc.* **1985**, 119–132 (1985).

37. L. M. Malard, M. A. Pimenta, G. Dresselhaus, M. S. Dresselhaus, Raman spectroscopy in graphene. *Phys. Rep.* **473**, 51–87 (2009).

38. D. G. Henry, I. Jarvis, G. Gillmore, M. Stephenson, Raman spectroscopy as a tool to determine the thermal maturity of organic matter: Application to sedimentary, metamorphic and structural geology. *Earth Sci. Rev.* **198**, 102936 (2019).

39. N. Ferralis, E. D. Matys, A. H. Knoll, C. Hallmann, R. E. Summons, Rapid, direct and non-destructive assessment of fossil organic matter via microRaman spectroscopy. *Carbon* **108**, 440–449 (2016).

40. L. J. Bellamy, *The Infra-red Spectra of Complex Molecules* (Chapman and Hall, 1975).

41. P. C. Painter, R. W. Snyder, M. Starsinic, M. M. Coleman, D. W. Kuehn, A. Davis, Concerning the application of FT-IR to the study of coal: A critical assessment of band assignments and the application of spectral analysis programs. *Appl. Spectrosc.* **35**, 475–485 (1981).

42. R. Lin, G. Patrick Ritz, Studying individual macerals using i.r. microspectrometry, and implications on oil versus gas/condensate proneness and “low-rank” generation. *Org. Geochem.* **20**, 695–706 (1993).

43. M. Dworkin, S. Falkow, E. Rosenberg, K.-H. Schleifer, E. Stackebrandt, *The Prokaryotes: Archaea. Bacteria: Firmicutes, Actinomycetes* (Springer, ed. 3, 2006), vol. 3.

44. E. Rosenberg, E. F. DeLong, S. Lory, E. Stackebrandt, F. Thompson, *The Prokaryotes: Other Major Lineages of Bacteria and the Archaea* (Springer, ed. 4, 2014).

45. S. C. Watkinson, L. Boddy, N. Money, *The Fungi* (Academic Press, ed. 3, 2016).

46. L. E. Graham, J. M. Graham, L. W. Wilcox, M. E. Cook, *Algae* (LJLM Press, ed. 3, 2016).

47. R. W. Castenholz, R. Rippka, M. Herdman, Form-genus IX. *Oscillatoria* in *Bergey's Manual® of Systematic Bacteriology, Volume One: The Archaea and the Deeply Branching and Phototrophic Bacteria*, D. R. Boone, R. W. Castenholz, Eds. (Springer, ed. 2, 2001), vol. 1, pp. 550–553.

48. R. Rippka, R. W. Castenholz, M. Herdman, Form-genus I. *Rivularia* in *Bergey's Manual® of Systematic Bacteriology, Volume One: The Archaea and the Deeply Branching and Phototrophic Bacteria*, D. R. Boone, R. W. Castenholz, Eds. (Springer, ed. 2, 2001), vol. 1, pp. 586–587.

49. E. L. Thurston, L. O. Ingram, Morphology and fine structure of Fischerella *ambigua1. J. Phycol.* **7**, 203–210 (1971).

50. R. W. Castenholz, R. Rippka, A. Wilmotte, Form-genus VII. *Lyngbya* in *Bergey's Manual® of Systematic Bacteriology, Volume One: The Archaea and the Deeply Branching and Phototrophic Bacteria*, D. R. Boone, R. W. Castenholz, Eds. (Springer, ed. 2, 2001), vol. 1, pp. 547–548.

51. W. R. Strohl, Genus III. *Beggiatoa* in *Bergey's Manual® of Systematic Bacteriology, Volume Two: The Proteobacteria, Part B: The Gammaproteobacteria*, D. J. Brenner, N. R. Krieg, J. T. Staley, Eds. (Springer, 2005), vol. 2, pp. 148–161.

52. B. B. Jørgensen, A. Teske, A. Ahmad, Genus VII. *Thioploca* in *Bergey's Manual® of Systematic Bacteriology, Volume Two: The Proteobacteria, Part B: The Gammaproteobacteria*, D. J. Brenner, N. R. Krieg, J. T. Staley, Eds. (Springer, 2005), vol. 2, pp. 171–178.

53. C. L. Thompson, R. Vier, A. Mikaelyan, T. Wienemann, A. Brune, '*Candidatus* Arthromitus' revised: Segmented filamentous bacteria in arthropod guts are members of Lachnospiraceae. *Environ. Microbiol.* **14**, 1454–1465 (2012).

54. J.-M. Volland, S. Gonzalez-Rizzo, O. Gros, T. Tyml, N. Ivanova, F. Schulz, D. Goudeau, N. H. Elisabeth, N. Nath, D. Udwary, R. R. Malmstrom, C. Guidi-Rontani, S. Bolte-Kluge, K. M. Davies, M. R. Jean, J.-L. Mansot, N. J. Mouncey, E. R. Angert, T. Woyke, S. V. Date, A centimeter-long bacterium with DNA contained in metabolically active, membrane-bound organelles. *Science* **376**, 1453–1458 (2022).

55. L. A. Hanic, Life history studies on Urospora and Codiolum from southern British Columbia, thesis, University of British Columbia (1965).

56. P. Kornmann, Ein Beitrag zur Taxonomie der *Gattung Chaetomorpha* (Cladophorales, Chlorophyta). *Helgolander Wiss. Meeresunters* **23**, 1–31 (1972).

57. A. H. Knoll, D. Lahr, Fossils, feeding, and the evolution of complex multicellularity in *Multicellularity, Origins and Evolution, The Vienna Series in Theoretical Biology: Boston,*

Massachusetts Institute of Technology, K. J. Niklas, S. A. Newman, Eds. (The MIT Press, 2016), pp. 1–16.

58. Y. Heilig, K. Schmitt, S. Seiler, Phospho-regulation of the *Neurospora crassa* septation initiation network. *PLOS ONE* **8**, e79464 (2013).

59. M. W. Dick, Straminipilous Fungi (Springer, 2001).

60. J. W. Brown, U. Sorhannus, A molecular genetic timescale for the diversification of autotrophic Stramenopiles (Ochrophyta): Substantive underestimation of putative fossil ages. *PLOS One* **5**, e12759 (2010).

61. N. H. Matari, J. E. Blair, A multilocus timescale for oomycete evolution estimated under three distinct molecular clock models. *BMC Evol. Biol.* **14**, 101 (2014).

62. F. Lutzoni, M. D. Nowak, M. E. Alfaro, V. Reeb, J. Miadlikowska, M. Krug, A. E. Arnold, L. A. Lewis, D. L. Swofford, D. Hibbett, K. Hilu, T. Y. James, D. Quandt, S. Magallón, Contemporaneous radiations of fungi and plants linked to symbiosis. *Nat. Commun.* **9**, 5451 (2018).

63. J. F. H. Strassert, I. Irisarri, T. A. Williams, F. Burki, A molecular timescale for eukaryote evolution with implications for the origin of red algal-derived plastids. *Nat. Commun.* **12**, 1879 (2021).

64. T. N. Taylor, M. Krings, E. L. Taylor, *Fossil Fungi* (Academic Press, Elsevier, 2015).

65. P. Sánchez-Baracaldo, J. A. Raven, D. Pisani, A. H. Knoll, Early photosynthetic eukaryotes inhabited low-salinity habitats. *Proc. Natl. Acad. Sci.* **114**, E7737–E7745 (2017).

66. H. C. Betts, M. N. Puttick, J. W. Clark, T. A. Williams, P. C. J. Donoghue, D. Pisani, Integrated genomic and fossil evidence illuminates life’s early evolution and eukaryote origin. *Nat. Ecol. Evol.* **2**, 1556–1562 (2018).

67. L. W. Parfrey, D. J. G. Lahr, A. H. Knoll, L. A. Katz, Estimating the timing of early eukaryotic diversification with multigene molecular clocks. *Proc. Natl. Acad. Sci.* **108**, 13624–13629 (2011).

68. N. J. Butterfield, Early evolution of the Eukaryota. *Palaeontology* **58**, 5–17 (2015).

69. S. M. Porter, Insights into eukaryogenesis from the fossil record. *Interface Focus* **10**, 20190105 (2020).

70. P. A. Cohen, R. B. Kodner, The earliest history of eukaryotic life: Uncovering an evolutionary story through the integration of biological and geological data. *Trends Ecol. Evol.* **37**, 246–256 (2022).

71. J. J. Brocks, A. J. M. Jarrett, E. Sirantoine, C. Hallmann, Y. Hoshino, T. Liyanage, The rise of algae in Cryogenian oceans and the emergence of animals. *Nature* **548**, 578–581 (2017).

72. S. Zhang, J. Su, S. Ma, H. Wang, X. Wang, K. He, H. Wang, D. E. Canfield, Eukaryotic red and green algae populated the tropical ocean 1400 million years ago. *Precambrian Res.* **357**, 106166 (2021).

73. J. J. Brocks, B. J. Nettersheim, P. Adam, P. Schaeffer, A. J. M. Jarrett, N. Güneli, T. Liyanage, L. M. van Maldegem, C. Hallmann, J. M. Hope, Lost world of complex life and the late rise of the eukaryotic crown. *Nature* **618**, 767–773 (2023).

74. E. J. Javaux, K. Lepot, The Paleoproterozoic fossil record: Implications for the evolution of the biosphere during Earth's middle-age. *Earth Sci. Rev.* **176**, 68–86 (2018).

75. L. Yin, X. Yuan, F. Meng, J. Hu, Protists of the Upper Mesoproterozoic Ruyang group in Shanxi Province, China. *Precambrian Res.* **141**, 49–66 (2005).

76. L. Y. Miao, M. Moczydlowska, S. X. Zhu, M. Y. Zhu, New record of organic-walled, morphologically distinct microfossils from the late Paleoproterozoic Changcheng group in the Yanshan Range, North China. *Precambrian Res.* **321**, 172–198 (2019).

77. T. N. Hermann, B. V. Timofeev, Eosolenides, a new group of problematic organisms from the Late Precambrian in *Problematics of the Late Precambrian and Paleozoic, Novosibirsk* (Nauka, 1985), pp. 9–15.

78. T. V. Jankauskas, N. S. Mikhailova, T. N. German, V. N. Sergeev, Z. M. Abduazimova, M. Y. Velova, M. B. Burzin, A. F. Veis, N. A. Volkova, V. K. Golovionok, A. Y. Grigorijeva, V. V.

Kirjanov, Y. V. Kozlova, P. N. Kolosov, L. N. Kraskov, I. N. Krylov, V. A. Luchinina, A. M. Medvedeva, R. N. Oqurizova, L. T. Paskiavichene, V. G. Piatiletov, V. A. Rudavskaya, I. A. Siverizeva, A. M. Stanevich, A. A. Treshchetenkova, Z. K. Faizulina, I. K. Chepikova, V. Y. Shenfil, E. D. Shepeleva, M. S. Yakshin, *Precambrian Microfossils of the USSR* (Nauka, 1989).

79. S. M. Porter, R. Meisterfeld, A. H. Knoll, Vase-shaped microfossils from the Neoproterozoic Chuar group, Grand Canyon: A classification guided by modern testate amoebae. *J. Paleo.* **77**, 409–429 (2003).

80. S. Tian, *Stratigraphy (Lithostratic) of the Municipality of Tianjin* (Multiple classification and correlation of the stratigraphy of China China Univ. of Geosciences Press, 1996), vol. 12.

81. H. Li, W. Su, H. Zhou, J. Geng, Z. Xiang, Y. Cui, W. Liu, S. Lu, The base age of the Changchengian system at the northern North China Craton should be younger than 1670 Ma: Constraints from zircon U-Pb LA-MC-ICP-MS dating of a granite-porphyry dike in Miyun County, Beijing. *Earth Sci. Front.* **18**, 108–120 (2011).

82. C. Duan, Y. Li, Y. Yang, Y. Liang, M. Wei, K. Hou, U-Pb ages and Hf isotopes of detrital zircon grains from the Mesoproterozoic Chuanlinggou Formation in North China Craton: Implications for the geochronology of sedimentary iron deposits and crustal evolution. *Minerals* **8**, 547 (2018).

83. S. Zhang, Y. Zhao, H. Ye, J. Hu, New constraints on ages of the Chuanlinggou and Tuanshanzi formations of the Changcheng system in the Yan-Liao area in the northern North China craton. *Acta Petrol. Sin.* **29**, 2481–2490 (2013).

84. L. Gao, C. Zhang, X. Shi, B. Song, Z. Wang, Y. Liu, Mesoproterozoic age for Xiamaling Formation in North China plate indicated by zircon SHRIMP dating. *Chin. Sci. Bull.* **53**, 2665–2671 (2008).

85. L. Gao, C. Zhang, C. Yin, X. Shi, Z. Wang, Y. Liu, P. Liu, F. Tang, B. Song, SHRIMP zircon ages: Basis for refining the chronostraigraphic classification of the Meso- and Neoproterozoic strata in North China old land. *Acta Geosci. Sin.* **29**, 366–376 (2008).

86. H. Li, S. Zhu, Z. Xiang, W. Su, S. Lu, H. Zhou, J. Geng, S. Li, F. Yang, Zircon U-Pb dating on tuff bed from Gaoyuzhuang Formation in Yanqing, Beijing: Further constraints on the new subdivision of the Mesoproterozoic stratigraphy in the northern North China craton. *Acta Petrol. Sin.* **26**, 2131–2140 (2010).

87. H. Li, W. Su, H. Zhou, Z. Xiang, H. Tian, L. Yang, W. D. Huff, F. R. Ettensohn, The first precise age constraints on the Jixian system of the Meso- to Neoproterozoic standard section of China: SHRIMP zircon U-Pb dating of bentonites from the Wumishan and Tieling formations in the Jixian section North China craton. *Acta Petrol. Sin.* **30**, 2999–3012 (2014).

88. E. R. Parodi, E. J. Cáceres, Life history and cytology of the freshwater alga *Chaetomorpha exilissima* sp. nov (Cladophorales, Chlorophyta). *Cytologia* **61**, 179–188 (1996).

89. F. Leliaert, D. A. Payo, H. P. Calumpong, O. De Clerck, *Chaetomorpha philippinensis* (Cladophorales, Chlorophyta), a new marine microfilamentous green alga from tropical waters. *Phycologia* **50**, 384–391 (2011).

90. B. Huang, L. Teng, L. Ding, Morphological and molecular discrimination of green macroalgae *Chaetomorpha aerea* and *C. linum*. *Acta Oceanol. Sin.* **35**, 118–123 (2016).

91. S. A. Felisberto, D. B. da Silva e Souza, Characteristics and diversity of cyanobacteria in Periphyton from Lentic tropical ecosystem, Brazil. *Adv. Appl. Microbiol.* **4**, 1076–1087 (2014).

92. V. U. Rani, U. E. Perumal, S. Palanivel, Morphology and taxonomy of *Oscillatoria princeps* Vaucher ex gomont (Oscillatoriales, Oscillatoriaceae). *Indian J. Edu. Inf. Manage.* **5**, 1–5 (2016).

93. R. Muhlsteinova, T. Hauer, P. De Ley, N. Pietrasiak, Seeking the true *Oscillatoria*: A quest for a reliable phylogenetic and taxonomic reference point. *Preslia* **90**, 151–169 (2018).

94. D. C. Nelson, C. O. Wirsen, H. W. Jannasch, Characterization of large, autotrophic *Beggiatoa* spp. abundant at hydrothermal vents of the Guaymas Basin. *Appl. Environ. Microbiol.* **55**, 2909–2917 (1989).

95. J. Larkin, M. C. Henk, P. Aharon, *Beggiatoa* in microbial mats at hydrocarbon vents in the Gulf of Mexico and warm mineral springs, Florida. *Geo-Marine Letters* **14**, 97–103 (1994).

96. J. M. Larkin, M. C. Henk, Filamentous sulfide-oxidizing bacteria at hydrocarbon seeps of the Gulf of Mexico. *Microsc. Res. Tech.* **33**, 23–31 (1996).

97. S. Hinck, T. R. Neu, G. Lavik, M. Mussmann, D. de Beer, H. M. Jonkers, Physiological adaptation of a nitrate-storing *Beggiatoa* sp. to diel cycling in a phototrophic hypersaline mat. *Appl. Environ. Microbiol.* **73**, 7013–7022 (2007).

98. J. P. de Albuquerque, C. N. Keim, U. Lins, Comparative analysis of *Beggiatoa* from hypersaline and marine environments. *Micron* **41**, 507–517 (2010).

99. M. R. N. Jean, S. Gonzalez-Rizzo, P. Gauffre-Autelin, S. K. Lengger, S. Schouten, O. Gros, Two new *Beggiatoa* species inhabiting marine mangrove sediments in the Caribbean. *PLOS One* **10**, e0117832 (2015).

100. S. Maier, V. A. Gallardo, *Thioploca araucae* sp. nov. and *Thioploca chileae* sp. nov. *Int. J. Syst. Evol. Microbiol.* **34**, 414–418 (1984).

101. M. Huettel, S. Forster, S. Kloser, H. Fossing, Vertical migration in the sediment-dwelling sulfur bacteria *Thioploca* spp. in overcoming diffusion limitations. *Appl. Environ. Microbiol.* **62**, 1863–1872 (1996).

102. S. Otte, J. G. Kuenen, L. P. Nielsen, H. W. Paerl, J. Zopfi, H. N. Schulz, A. Teske, B. Strotmann, V. A. Gallardo, B. B. Jørgensen, Nitrogen, carbon, and sulfur metabolism in natural *Thioploca* samples. *Appl. Environ. Microbiol.* **65**, 3148–3157 (1999).

103. H. N. Schulz, B. Strotmann, V. A. Gallardo, B. B. Jørgensen, Population study of the filamentous sulfur bacteria *Thioploca* spp. off the Bay of Concepcion, Chile. *Mar. Ecol. Prog. Ser.* **200**, 117–126 (2000).

104. P. J. Keeling, F. Burki, Progress towards the tree of eukaryotes. *Curr. Biol.* **29**, R808–R817 (2019).

105. F. Burki, A. J. Roger, M. W. Brown, A. G. B. Simpson, The new tree of eukaryotes. *Trends Ecol. Evol.* **35**, 43–55 (2020).
